# Synthesis of 1,2,4-Oxadiazole Sulfonamide Derivatives and Their Biological Investigation as Monoamine Oxidase Inhibitors

**DOI:** 10.3390/molecules31183326

**Published:** 2026-09-19

**Authors:** Anton A. Shetnev, Olga A. Gasilina, Sergey V. Baykov, Rakhymzhan Turmanov, Nurbol Appazov, Rakhmetulla Zhapparbergenov, Nurila Togyzbayeva, Mikhail K. Korsakov, Stephanus J. Cloete, Anél Petzer, Jacobus P. Petzer

**Affiliations:** 1 Institute of Chemistry, Saint Petersburg State University, Universitetskaya Nab., 7/9, 199034 Saint Petersburg, Russia; a.shetnev@yspu.org (A.A.S.); sergei.v.baikov@yandex.ru (S.V.B.); mkkors@mail.ru (M.K.K.); 2 Pharmaceutical Technology Transfer Centre, Yaroslavl State Pedagogical University Named After K. D. Ushinsky, Respublikanskaya St., 108, 150000 Yaroslavl, Russia; olgagasilina23@yandex.ru; 3 Laboratory of Engineering Profile “Physical and Chemical Methods of Analysis”, Korkyt Ata Kyzylorda University, 29 Aiteke bi Str., Kyzylorda 120000, Kazakhstan; nurasar.82@korkyt.kz (N.A.); ulagat-91@mail.ru (R.Z.); nurila2009@mail.ru (N.T.); 4 “DPS Kyzylorda” LLP, Amangeldy Imanov Str., 112A, Kyzylorda 120008, Kazakhstan; 5 “CNEC’’ LLP, Dariger Ali Lane, 2, Kyzylorda 120001, Kazakhstan; 6 Pharmaceutical Chemistry and Centre of Excellence for Pharmaceutical Sciences, North-West University, Potchefstroom 2520, South Africa; 23496959@mynwu.ac.za (S.J.C.); anel.petzer@nwu.ac.za (A.P.)

**Keywords:** monoamine oxidase, MAO, oxadiazole, sulfonamide, inhibition, Parkinson’s disease

## Abstract

Monoamine oxidase (MAO) enzymes catalyze the catabolism of neurotransmitter amines, and MAO inhibitors are therefore of much clinical value. Based on the continued interest in MAO inhibitors, the present study examined the MAO inhibition properties of 1,2,4-oxadiazole sulfonamide derivatives. While we previously investigated benzenesulfonamides as MAO inhibitors, the incorporation of the 1,2,4-oxadiazole ring as the heteroaromatic ring is novel. 1,2,4-Oxadiazole sulfonamide derivatives were prepared from the corresponding carboxylic acids and amidoximes by an acylation–cyclization reaction under basic conditions, and MAO inhibition properties were determined using recombinant human enzymes. The derivatives potently inhibited MAO-B, with seven compounds exhibiting IC_50_ values lower than 0.02 µM. In contrast, comparatively weak MAO-A inhibition was observed with IC_50_ values greater than 6.04 µM. Mechanistic studies indicated that a representative compound acted as a reversible and competitive inhibitor. It may be concluded that 1,2,4-oxadiazole sulfonamide derivatives exhibit promising, isoform-selective MAO-B inhibition that may be of value for future therapeutic application.

## 1. Introduction

Monoamine oxidase (MAO) enzymes are flavin adenine dinucleotide (FAD)-dependent amine oxidases that catalyze the oxidative deamination of key neurotransmitters such as serotonin, norepinephrine and dopamine as well as dietary amines such as tyramine and phenethylamine [1]. These enzymes are validated drug targets, and their inhibitors are employed in the management of Parkinson’s disease and depression [2,3,4]. MAO-A inhibitors exert an antidepressant effect by enhancing the brain levels of serotonin and norepinephrine, while in Parkinson’s disease, MAO-B inhibitors provide symptomatic relief from motor symptoms by increasing striatal dopamine levels [5,6]. Significantly, MAO-B inhibitors may also possess neuroprotective properties by reducing the formation of hydrogen peroxide during MAO catalytic turnover, which subsequently decreases oxidative damage to neuronal tissue [7].

MAOs are also of interest in several other disease states. MAO-A activity in the cardiac muscle has been associated with oxidative stress leading to mitochondrial damage, and MAO-A inhibitors may therefore have a future role in the treatment of cardiovascular disease [8,9,10]. In prostate cancer, MAO-A has been found to promote prostate cancer progression, while the inhibition of MAO-A activity reduced xenograft growth in experimental animals [11,12]. Other areas where MAO inhibitors may have future therapeutic roles are rheumatoid arthritis and neuropathic pain [13,14,15]. In rheumatoid arthritis, MAO inhibitors may counter the progression of the disease by reducing catecholamine breakdown and hydrogen peroxide formation in synovial fluid [14]. MAO-B inhibitors have shown potential to suppress neuroinflammation, while it was recently demonstrated that upregulated MAO-B in reactive astrocytes contributes to neuroinflammation in Alzheimer’s disease, opening new avenues for MAO-B inhibitors in this condition [16,17]. Furthermore, MAO promotes amyloid-beta (Aβ) deposition and contributes to the generation of neurofibrillary tangles and cognitive impairment, which further underscore a role for MAO inhibitors in Alzheimer’s disease [16]. Clinically approved MAO inhibitors include both irreversible (e.g., tranylcypromine, phenelzine, rasagiline, selegiline) and reversible (e.g., moclobemide, safinamide) agents [1]. The highly selective reversible MAO-B inhibitor, tisolagiline (KDS2010), has successfully completed Phase I clinical trials and is currently under development for the treatment of Alzheimer’s disease and obesity [18].

**Figure 1:**
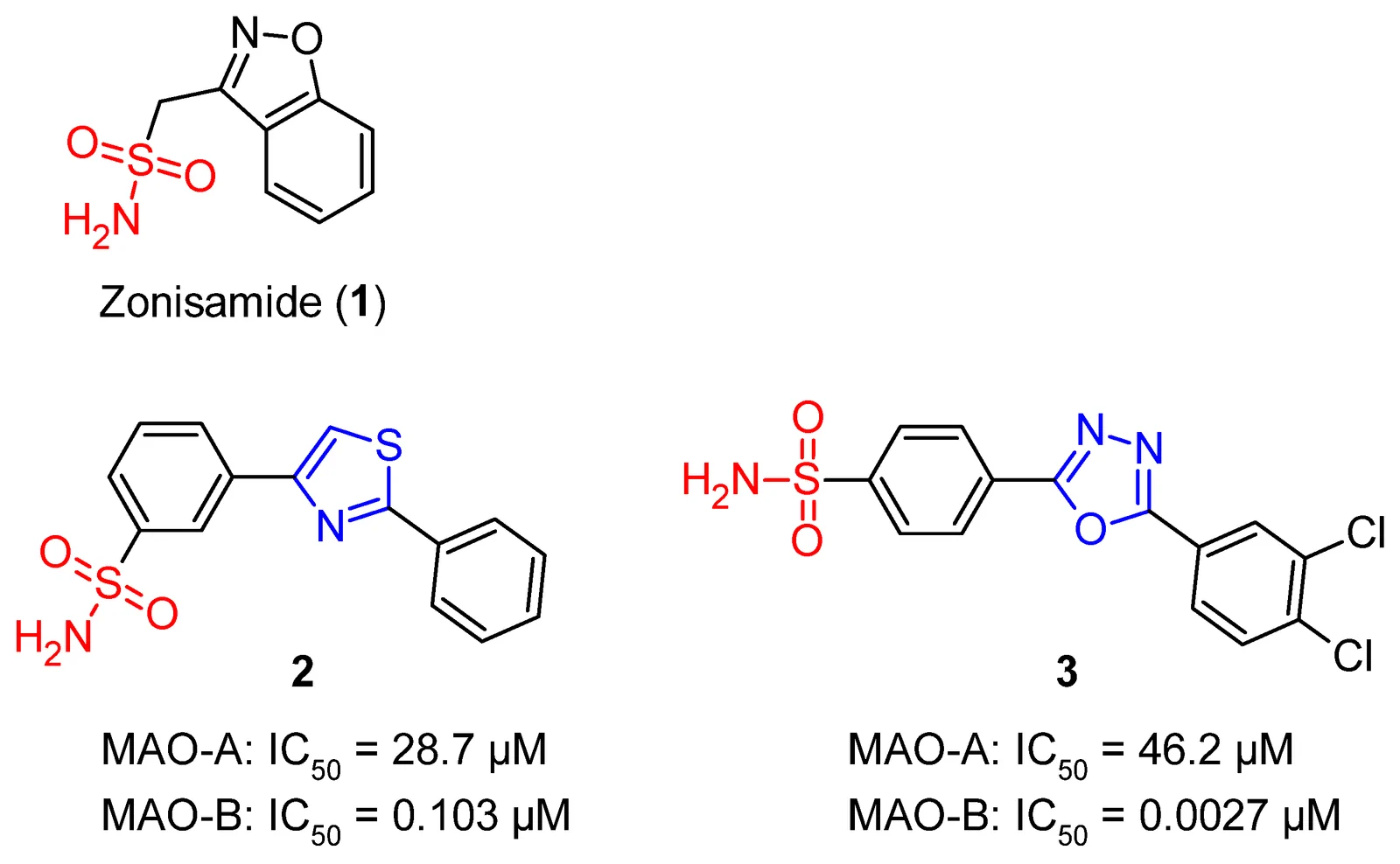
The structures of zonisamide (**1**) and benzenesulfonamide compounds (**2** and **3**) that have been reported to inhibit MAO-B.

Current efforts in MAO inhibitor development are directed towards improved isoform selectivity, enhanced safety profiles, and multi-target compounds that combine MAO inhibition with neuroprotective, antioxidant or metal-chelating properties. Reversible and MAO-B-selective inhibitors that minimize the risk of hypertensive tyramine-induced reactions are of particular interest. Based on the continued interest in MAO inhibition as a therapeutic strategy, the current study synthesized and investigated a series of 1,2,4-oxadiazole sulfonamide derivatives [19,20]. Sulfonamide derivatives represent a promising class for the design of MAO-B inhibitors. One of the first examples of an MAO inhibitor within this class was zonisamide (**1**), a heterocyclic compound substituted with a primary sulfonamide group (Figure 1) [21]. Zonisamide is an anticonvulsant that is also used as an adjunct to levodopa for the treatment of the motor symptoms of Parkinson’s disease [22,23]. Zonisamide was reported to be an MAO-B-selective inhibitor with an IC_50_ value of 24.8 µM [21]. The elucidation of the interactions of zonisamide with MAO-B was carried out by X-ray crystallography, which showed that zonisamide binds within the substrate cavity of the enzyme, leaving the entrance cavity unoccupied [24]. Specific interactions included hydrogen bonding with the side chain of Gln206 and two conserved water molecules, of which one bridges Lys296 to the N(5) of the FAD. We subsequently discovered that several heterocyclic benzenesulfonamide derivatives exhibit potent and isoform-selective MAO inhibition. In previous studies, we designed a series of benzenesulfonamides based on the pyridazine, quinoline, 1,3-oxazole, 1,3-thiazole, and 1,3,4-oxadiazole structural motifs [25,26,27,28,29,30]. Among the benzenesulfonamides, several high-potency MAO-B inhibitors were discovered, as exemplified by compounds **2** (IC_50_ = 0.103 µM) and **3** (IC_50_ = 0.0027 µM) [29,30]. 1,3,4-Oxadiazole substituted benzenesulfonamide derivatives were found to possess the highest MAO-B inhibition potencies, with all reported compounds exhibiting submicromolar potencies [30]. It is noteworthy that no submicromolar MAO-A inhibitors were discovered among the benzenesulfonamides, indicating that this class exhibits selectivity for the MAO-B isoform.

**Figure 2:**
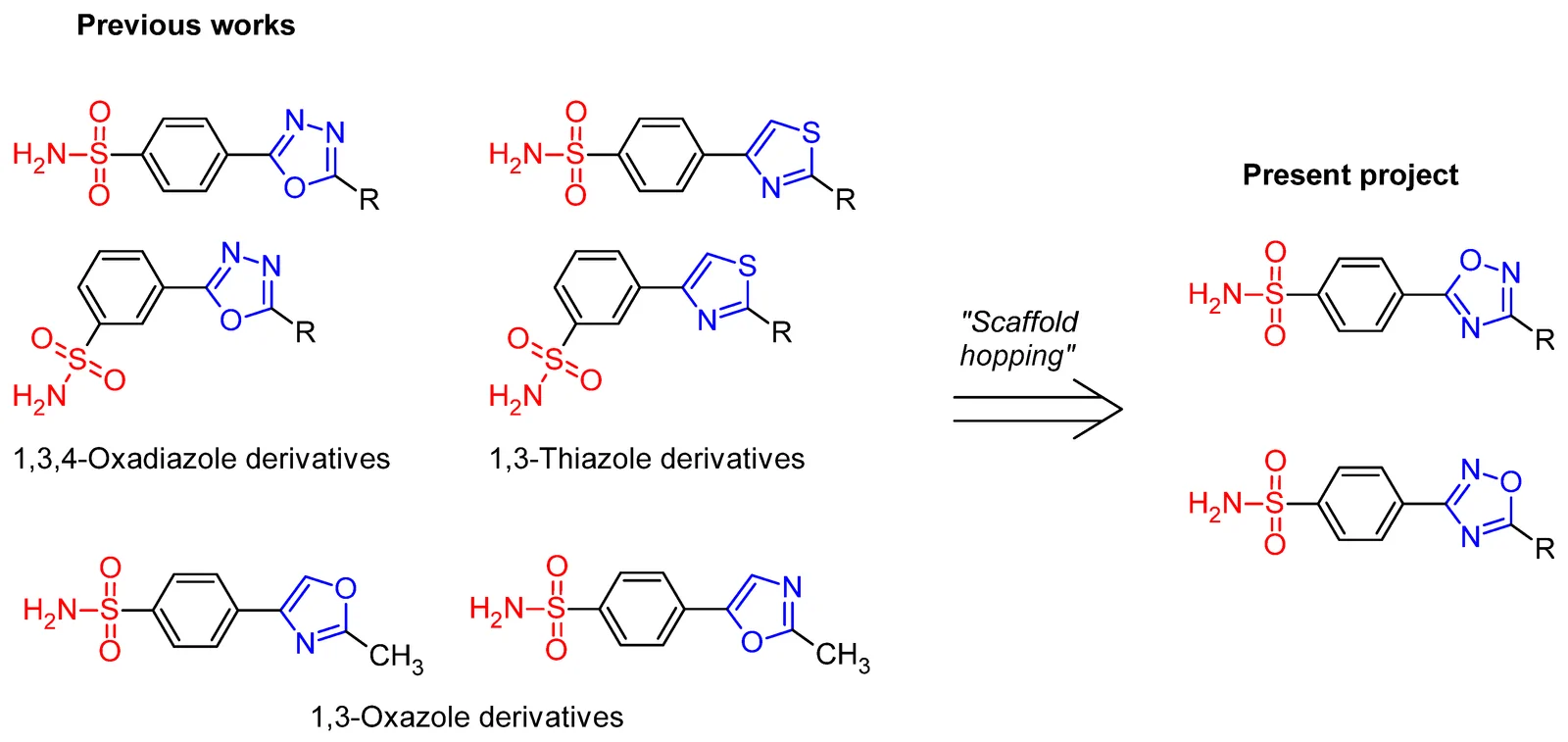
The approach that was followed for the design of 1,2,4-oxadiazole sulfonamide derivatives as potentially new MAO inhibitors.

In this study, using a scaffold hopping approach, we continued the search for novel MAO inhibitors among a series of 1,2,4-oxadiazole derivatives substituted with a primary arylsulfonamide moiety (Figure 2). These studies represent unique and novel contributions to scientific knowledge on the MAO inhibition properties of sulfonamide derivatives.

## 2. Results and Discussion

### 2.1. Chemistry

**Scheme 1:**
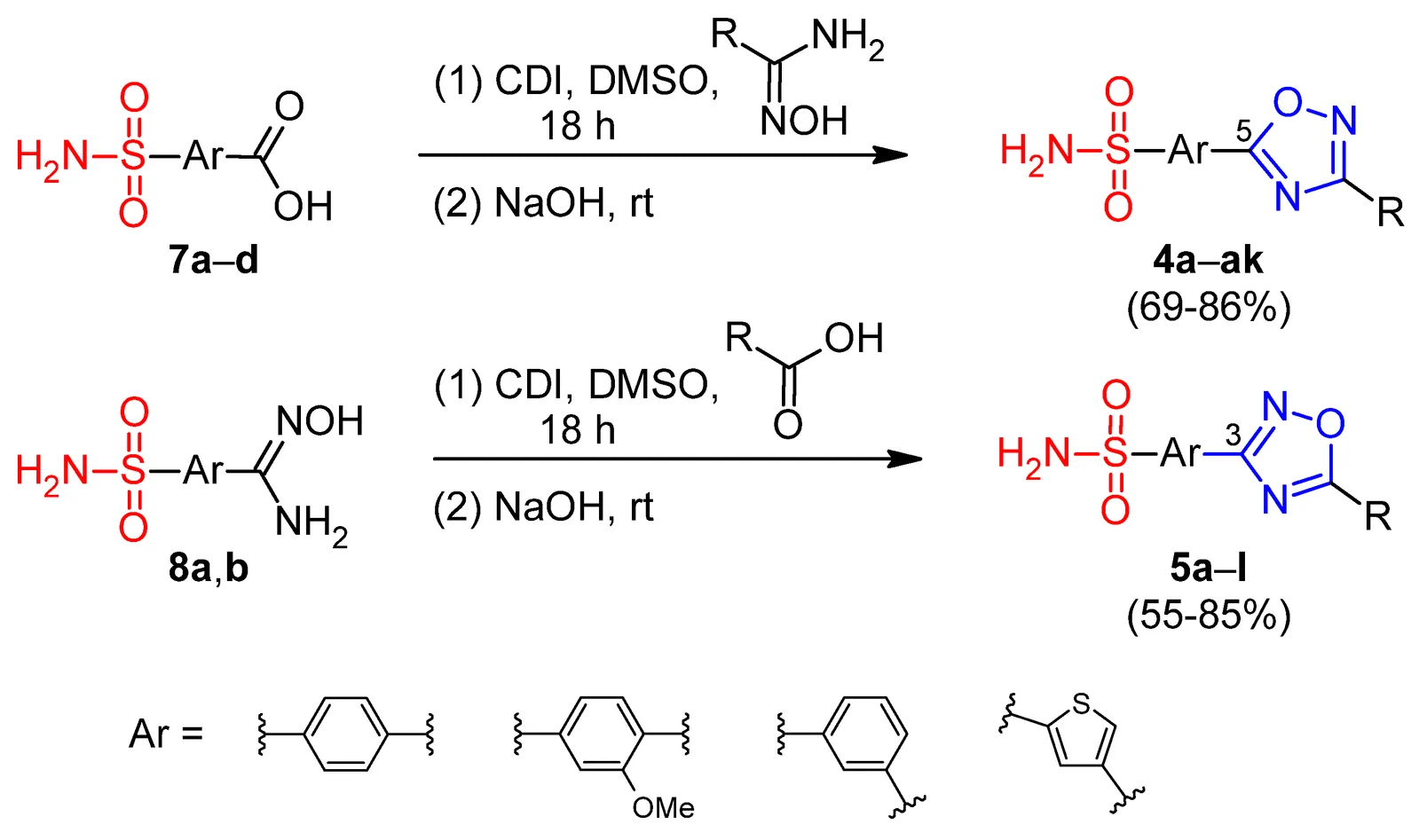
Preparation of 1,2,4-oxadiazole arylsulfonamide derivatives **4** and **5**.

The 1,2,4-oxadiazole sulfonamide derivatives **4a**–**ak** and **5a**–**l** were prepared from the corresponding carboxylic acids (**7**) and amidoximes (**8**) by an acylation–cyclization reaction under basic conditions with the participation of the coupling agent, 1,1′-carbonyldiimidazole (CDI), according to the method reported in the literature (Scheme 1) [31].

**Scheme 2:**
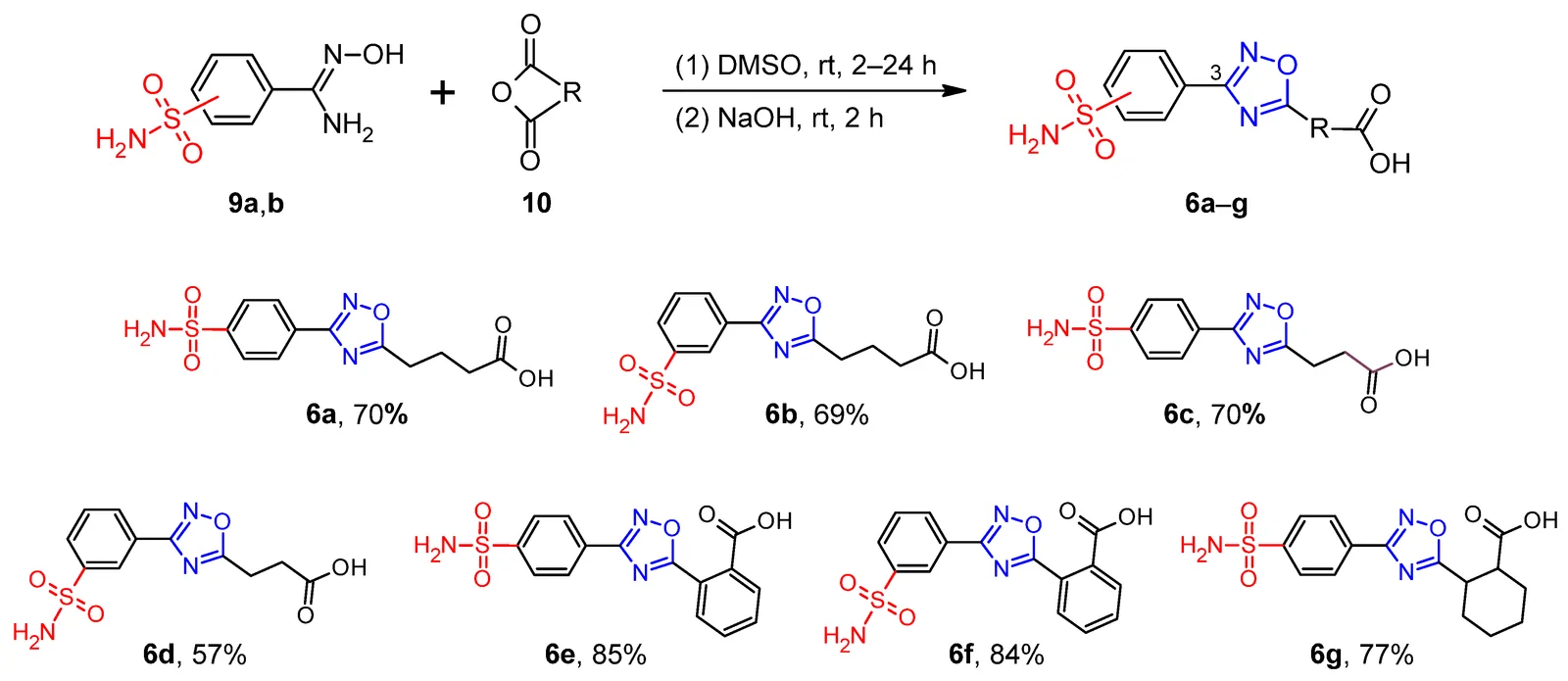
The preparation of 1,2,4-oxadiazole benzenesulfonamide derivatives (**6**) bearing the carboxyl group.

In addition, a series of 1,2,4-oxadiazole benzenesulfonamide derivatives bearing a free carboxylic acid group (**6**) was synthesized to increase polarity by introducing an ionizable group (Scheme 2). The literature procedure was followed, where benzenesulfonamide amidoxime **9** was acylated with the cyclic anhydride **10**, which was prepared from the corresponding dicarboxylic acid, and the resulting *O*-acylamidoxime was subsequently cyclized without isolation, also in a superbasic medium, which led to the formation of carboxylic acids **6a**–**g** with high to medium yields [32,33].

All synthesized compounds were fully characterized by ^1^H and ^13^C NMR spectroscopy and high-resolution mass spectrometry (HRMS). The obtained spectral data are in full agreement with the proposed structures. In particular, the ^1^H NMR spectra exhibit a characteristic singlet for the sulfonamide NH_2_ protons in the region of δ 7.5–7.6 ppm. In the ^13^C NMR spectra, the two carbon atoms of the 1,2,4-oxadiazole ring appear as distinct signals in the range of δ 157–179 ppm, depending on the substituents, which is consistent with literature precedents for such heterocycles. For compounds bearing a carboxylic acid group (series **6**), the carbonyl carbon signal is observed at δ 180–182 ppm for aliphatic acids and at δ ~168 ppm for benzoic acid derivatives. The HRMS data further confirm the molecular formulas of all target compounds. These observations collectively establish the identity and purity of the synthesized derivatives.

### 2.2. Biochemical Evaluation for MAO Inhibition

#### 2.2.1. MAO Inhibition Potencies

**Figure 3:**
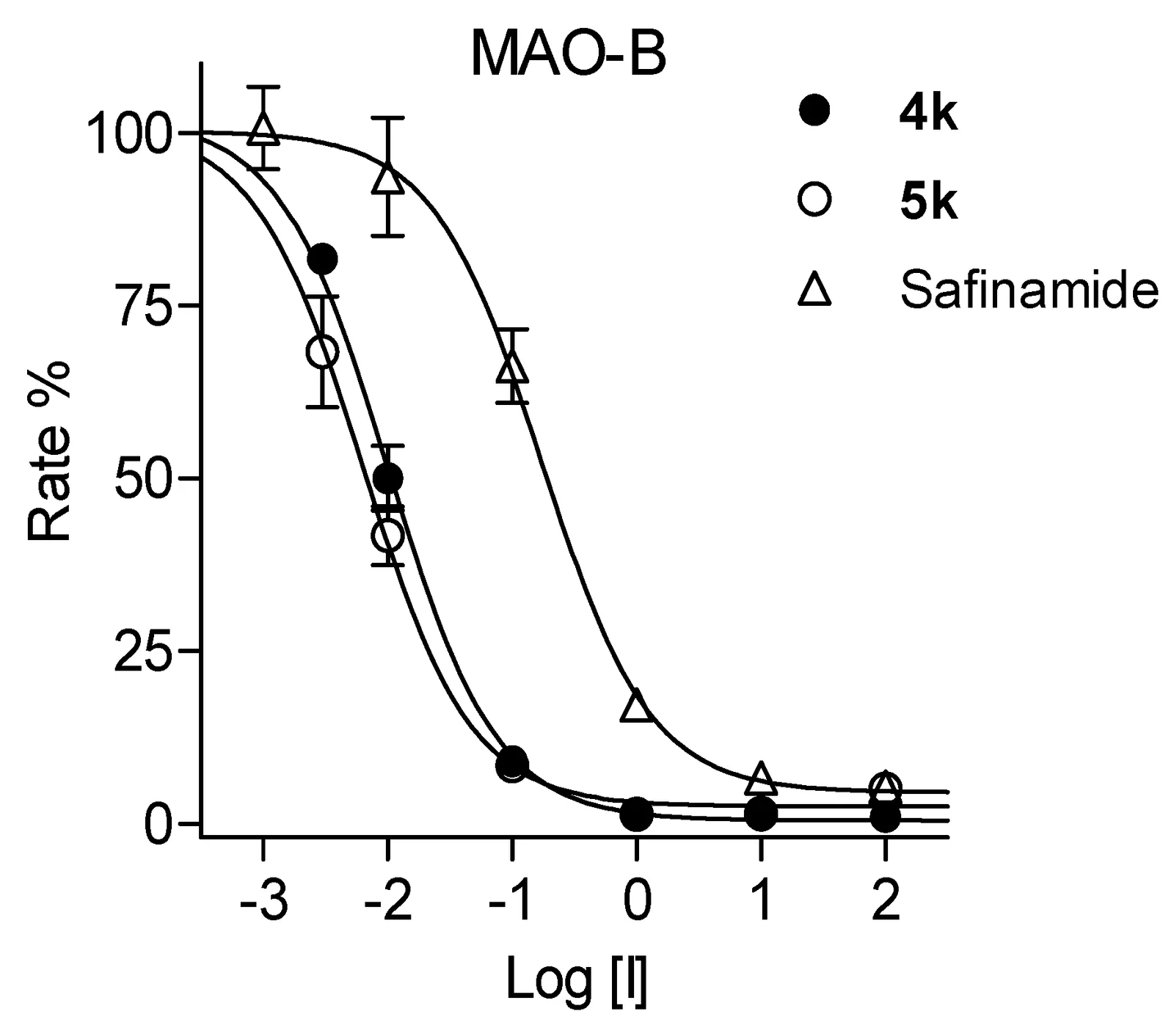
Examples of the inhibition graphs used to estimate the IC_50_ values of selected derivatives and a reference compound, safinamide, against MAO-B. The IC_50_ values for these derivatives were: **4k**, IC_50_ = 0.0079 µM; **5k**, IC_50_ = 0.0045 µM; safinamide, IC_50_ = 0.176 µM.

The primary sulfonamide derivatives **4a**–**ak**, **5a**–**l** and **6a**–**g** that were synthesized in this study were evaluated as potential in vitro inhibitors of human MAO-A and MAO-B according to the literature procedure (see Section 3) [30,34]. The inhibition potencies were expressed as the IC_50_ values, which are summarized in Table 1. Examples of the inhibition graphs used for the measurement of IC_50_ values are given in Figure 3.

The results show that the synthesized 1,2,4-oxadiazole sulfonamide derivatives functioned primarily as potent and highly selective inhibitors of the MAO-B isoform over MAO-A. Across all tested series, the majority of active compounds exhibited nanomolar to submicromolar inhibition against MAO-B while demonstrating minimal or no inhibition against MAO-A. The most potent MAO-A inhibitors were **5c** and **5d** with IC_50_ values of 6.04 and 6.99 µM, respectively. It is noteworthy that both these compounds were substituted on position 3 with the arylsulfonamide moiety as opposed to position 5 as was explored for other derivatives (e.g., **4a–ak**). Furthermore, these derivatives contained the *m*-phenylene linker. The most potent MAO-B inhibitors were derivatives **4k** (IC_50_ = 0.0079 µM) and **5k** (IC_50_ = 0.0045 µM). These were the only derivatives with an IC_50_ < 0.01 µM; however seven derivatives exhibited an IC_50_ < 0.02 µM (**4b**, **4c**, **4k**, **4l**, **4o**, **5h** and **5k**), and 14 derivatives exhibited an IC_50_ < 0.1 µM. These derivatives had similar inhibition potencies to the reference MAO-B inhibitor, safinamide (IC_50_ = 0.176 µM). This finding underscored the potent MAO-B inhibition that was observed for the series. While high-potency MAO-B inhibitors were recorded for compounds substituted with both substitution patterns of 1,2,4-oxadiazole (e.g., **4a–ak** vs. **5a–l**), the carboxylic acid containing derivatives (**6a–g**) were weak MAO inhibitors, which demonstrated the deleterious effect of this group for inhibition. Other structure–activity relationships (SARs) could also be derived from this study and are discussed below.

*Variation in the substituents and substitution patterns on the R-group*: Several substituted phenyl rings were evaluated as R-groups. While clear trends were not apparent, *meta*-methoxy substitution (**4f**) resulted in more potent MAO-B inhibition than *ortho*- (**4e**) or *para*-substitution (**4d**). However, *para*-methyl (**4b**) substitution was equipotent to *meta*-substitution (**4c**), while *meta*-chloro (**4k**) substitution was more favourable than *ortho*-substitution (**4j**). Similarly, *para*-fluoro (**4m**) substitution was similar in potency to *ortho*-substitution (**4o**), which was more favourable than *meta*-substitution (**4n**). A substituent on the phenyl was not a prerequisite since the unsubstituted compounds **4a** (IC_50_ = 0.097 µM), **5d** (IC_50_ = 0.095 µM) and **5e** (IC_50_ = 0.135 µM) also proved to be potent MAO-B inhibitors. It is noteworthy that both *para*-fluoro substituted-derivatives (**4m**, **5h**) were highly potent MAO-B inhibitors with an IC_50_ < 0.025 µM. However, substitution with various groups (e.g., F, Cl, Br, Me, OMe, CN) including t-butyl (**4r**) on the phenyl yielded potent MAO-B inhibitors, while disubstitution (**4g**, **5k**) was also effective.

*Variation in the R-group*: Heteroaromatic systems (e.g., thiophene, pyridyl) were also considered R-groups. As exemplified by **4u**, **4v**, **4w** and **4y,** 3-thiophene, 2-thiophene, 2-pyridyl and 4-pyridyl substitutions yielded good potency inhibition and were more favourable than the 3-pyridyl (**4x**)-substituted homologue. Interestingly, the phenylcarbamoyl (e.g., **4s**) and diphenylether (e.g., **4h**) groups also produced potent MAO-B inhibitors. Isopropyl (**4q**), cyclopropyl (**4p**, **5b**) and methyl (**4z**, **5g**) substitution on the other hand yielded weak or no MAO-B inhibition, indicating that larger R-groups are preferred.

*Variation in the linker*: This study also evaluated the effect of different linkers between the primary sulfonamide and 1,2,4-oxadiazole moieties. *para*-Phenylene (**4j**, **4x**) and *meta*-phenylene (**4ah**, **4ag**) substitution yielded similar inhibition potencies. For certain derivatives, the *meta*-phenylene linker (**5d**) led to higher-potency MAO-B inhibition than the *para*-phenylene linker (**5e**). Substitution with a methoxy group on the phenyl ring of the *para*-phenylene linker (e.g., **4aa**–**af**) was well tolerated and yielded good-potency MAO-B inhibition; however, apart from **4aa**, the corresponding unsubstituted homologues were more potent inhibitors. This demonstrated that methoxy substitution on the *para*-phenylene linker generally would not enhance MAO-B inhibition. Four derivatives containing a thiophene linker (**4ai**–**ak**, **5a**) were also studied. These derivatives were found to be weaker MAO-B inhibitors than the homologues containing the *para*-phenylene linker. This result shows that the optimization of both the linker and R-group will be required to obtain the combination that yields the highest inhibition potency. In this regard, the *para*-phenylene linker seems to be a suitable choice since it is present in the most potent inhibitors (IC_50_ < 0.02 µM) in this study.

Considering the MAO inhibition data for sulfonamide derivatives reported in the literature, a notable similarity with the present study is the selective inhibition of MAO-B compared with the MAO-A isoform [29,30]. This is exemplified by both compounds **2** and **3**. In line with this study, the literature also reports that carboxylic acid derivatives are generally weak MAO inhibitors [26]. The literature also indicates that introducing a phenyl substituent on the heteroaromatic ring markedly increases MAO-B inhibition and that adding a halogen to the phenyl ring is especially beneficial for MAO-B inhibitory activity. In this context, dichloro substitution has been shown in multiple cases to produce highly potent MAO-B inhibitors [29,30]. The current study indicates that the phenyl group can likewise be substituted with heteroaromatic ring systems (e.g., thiophene, pyridyl) while still maintaining potent MAO-B inhibitory activity.

#### 2.2.2. Reversibility and Mode of MAO Inhibition

**Figure 4:**
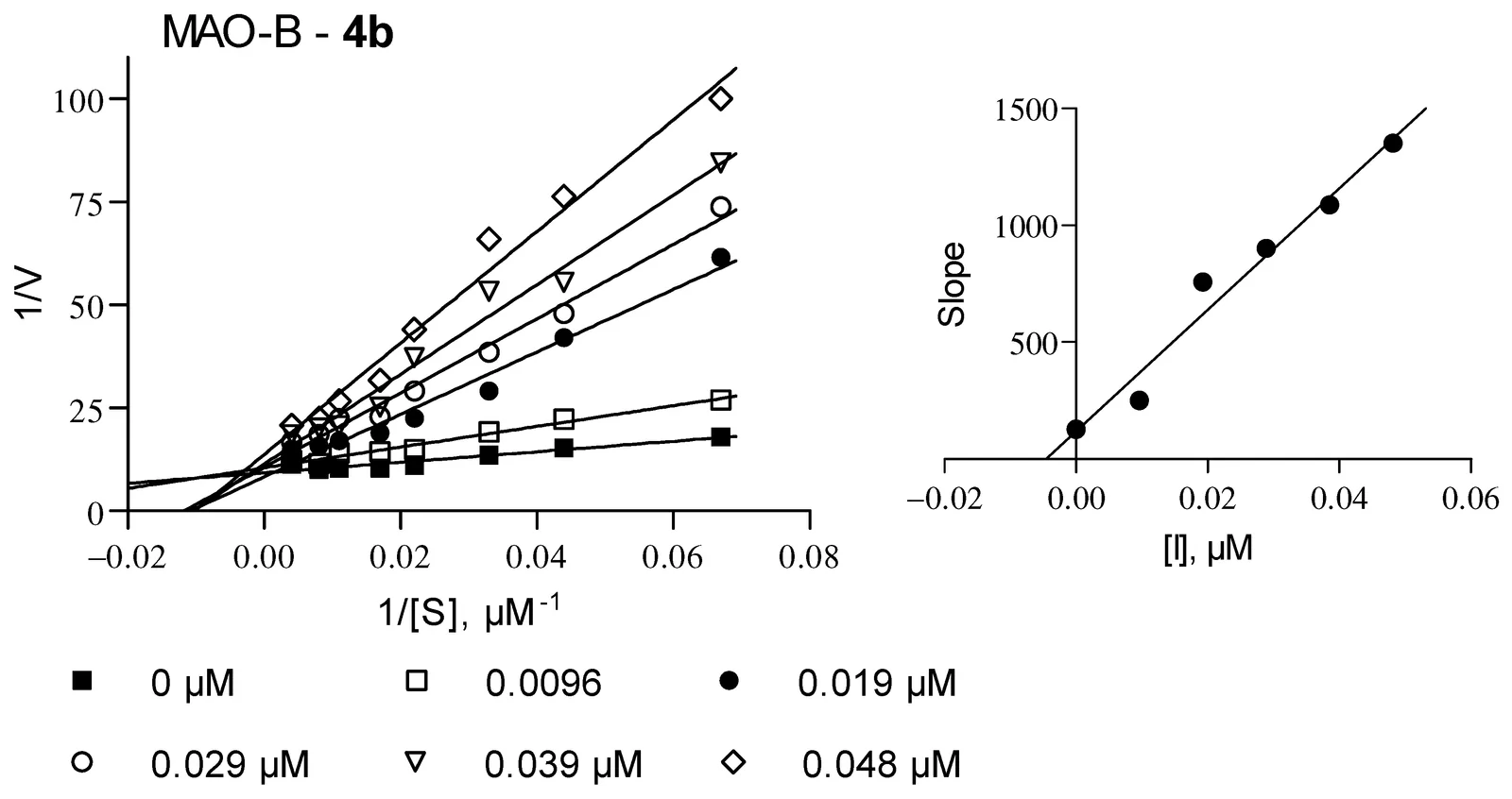
Lineweaver–Burk graphs for the inhibition of MAO-B by 1,2,4-oxadiazole sulfonamide derivative **4b**. The secondary graph is a plot of the slopes of the Lineweaver–Burk graphs versus inhibitor concentration and was used to estimate the K_i_ value.

This study found that numerous 1,2,4-oxadiazole sulfonamide derivatives were potent and selective MAO-B inhibitors. To investigate the mechanism of MAO-B inhibition, derivative **4b** was selected as a representative MAO-B inhibitor. This compound exhibited an IC_50_ value of 0.016 µM for the inhibition of MAO-B. The mode of inhibition was firstly investigated by preparing Lineweaver–Burk graphs (or double reciprocal plots) for the inhibition of MAO-B by **4b**. Five Lineweaver–Burk graphs were prepared at various inhibitor concentrations (0.0096–0.048 µM), and one graph was prepared in the absence of an inhibitor. The Lineweaver–Burk graphs are presented in Figure 4 and indicated that **4b** was a competitive MAO-B inhibitor since the lines intersected on the *y*-axis. The enzyme inhibitor dissociation constant (K_i_ value) was estimated from a secondary plot of the slopes of the Lineweaver–Burk graphs versus inhibitor concentration and was found to be 0.0046 µM, indicating high potency inhibition.

**Figure 5:**
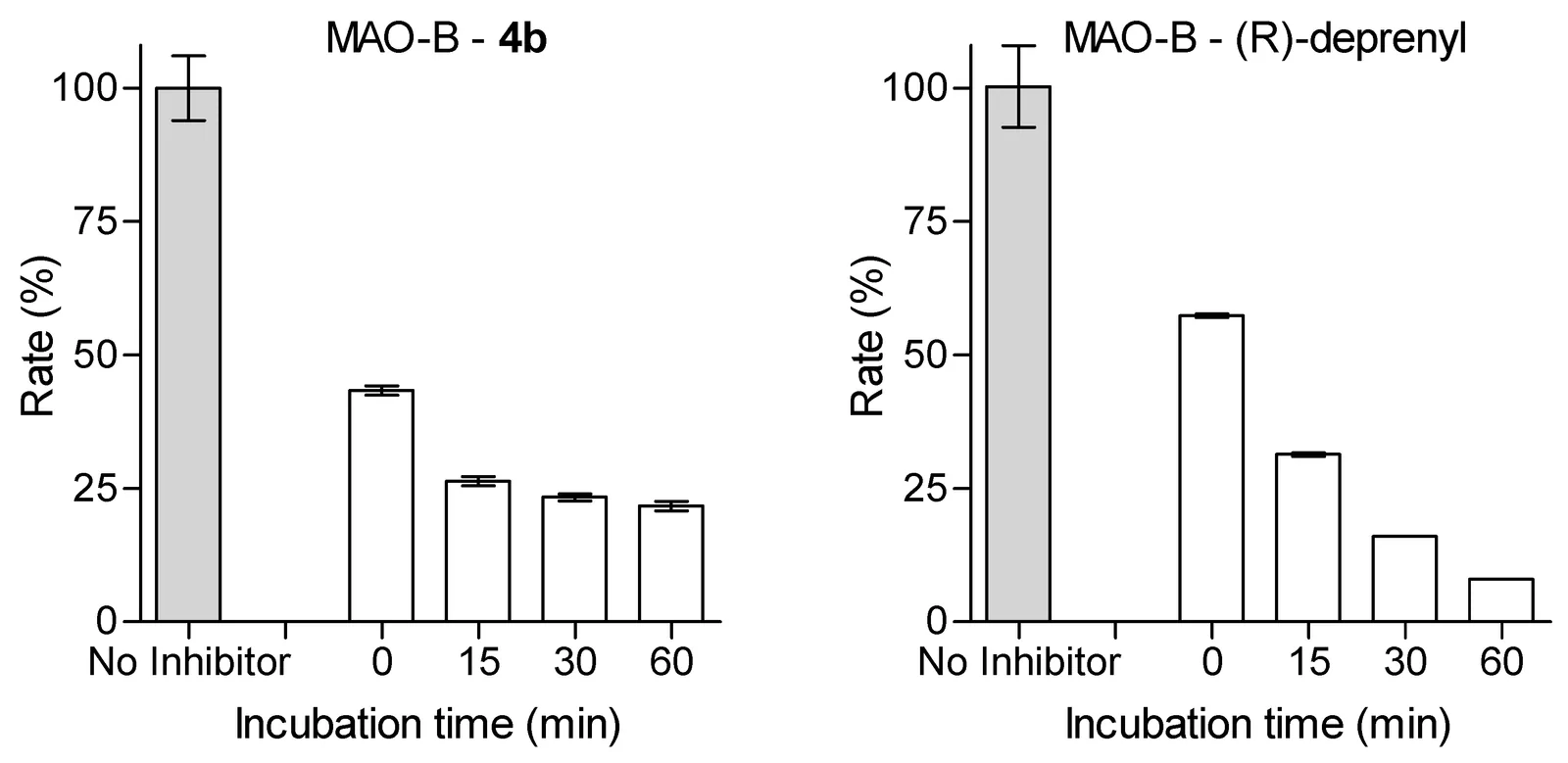
The time-dependent inhibition of MAO-B by 1,2,4-oxadiazole sulfonamide derivative **4b** and (R)-deprenyl. The inhibitors were co-incubated with MAO-B for various periods of time, and residual enzyme activity was measured. Each data point represents the mean (±standard deviation) of 3 technical replicates.

The observation that **4b** competitively inhibited MAO-B indicated that this derivative interacted reversibly with the enzyme. To verify this, the time dependency of inhibition was investigated. The MAO-B enzyme and **4b** were co-incubated for various time periods (0, 15, 30 and 60 min) with the concentration of **4b** being equal to 2 × IC_50_. After adding the enzyme substrate, kynuramine, and diluting the incubations to obtain an inhibitor concentration that was equal to IC_50_, the residual MAO-B activities were measured. The results are presented graphically in Figure 5 and show that after an initial reduction in activity from 0 to 15 min incubation, the MAO-B activities remained unchanged, which indicated that inhibition is not time-dependent and therefore reversible. In contrast, during a similar experiment with the irreversible MAO-B inhibitor, (R)-deprenyl, a clear reduction in enzyme activity was observed with increasing incubation time. These results confirmed that **4b** was indeed a reversible MAO-B inhibitor. The reversibility of inhibition agreed with the findings that sulfonamide compounds from previous studies also exhibited reversible MAO-B inhibition, while the derivatives do not possess the functional groups that are typically associated with the inactivation of MAO enzymes [21,30].

### 2.3. Molecular Docking

The X-ray crystal structure of zonisamide complexed to human MAO-B has been reported, which enabled a comparison with the binding modes of selected sulfonamide derivatives in the current study [24]. For this purpose, the two most potent MAO-B inhibitors, **4k** and **5k**, were selected and docked into the reported structure of zonisamide bound to MAO-B (PDB code: 3PO7). Docking was performed with the CDOCKER application of Discovery Studio 3.1 according to the protocol described recently [29,30]. Evidence that the protocol used for molecular docking is valid was provided by redocking the structure of zonisamide into the active site of MAO-B. A root mean square deviation (RMSD) of 1.47 Å was recorded, which indicated successful docking.

**Figure 6:**
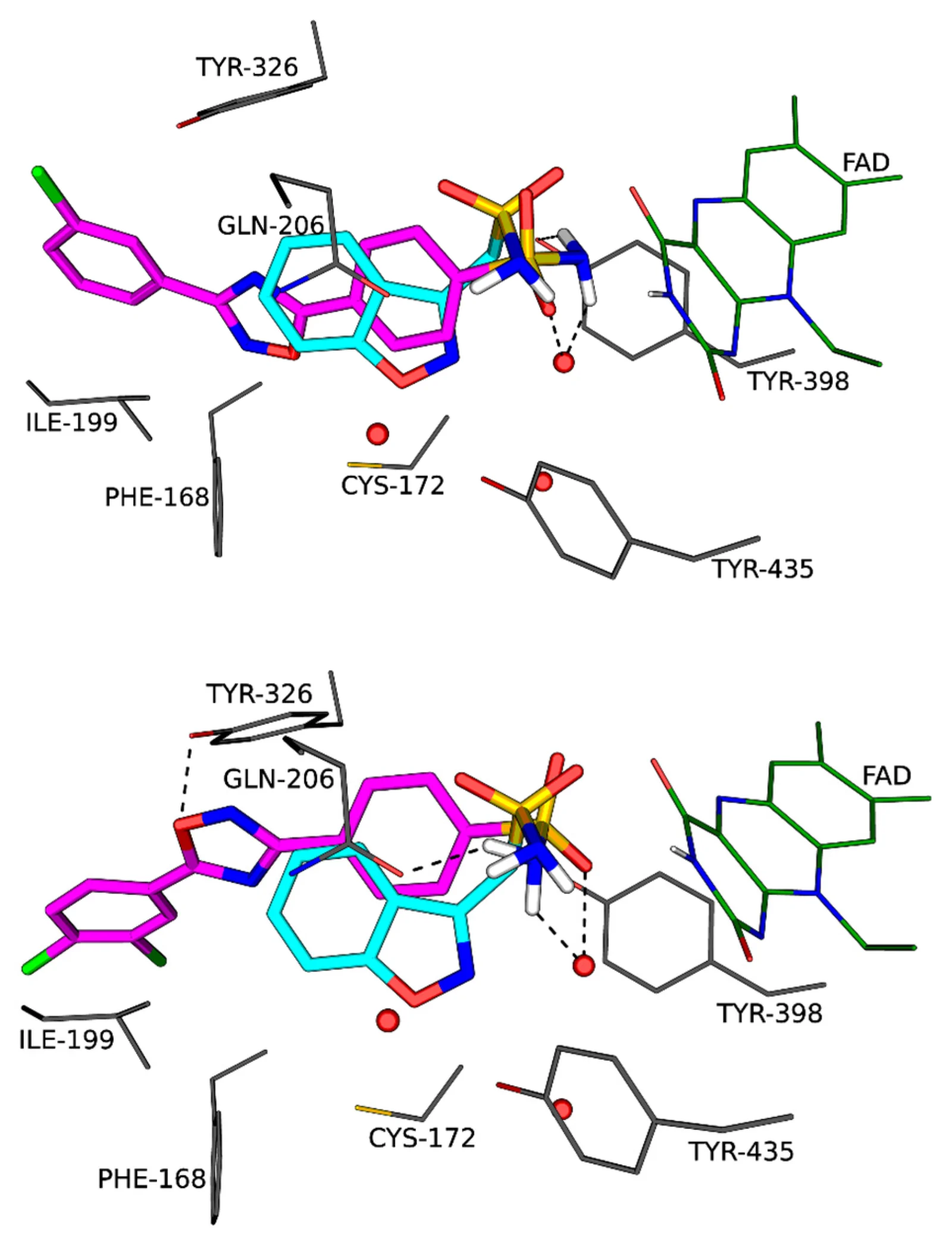
The predicted binding orientations of derivatives **4k** (**top**) and **5k** (**bottom**) to the active site of MAO-B. The inhibitors are shown in magenta, and hydrogen bond interactions are indicated by dashed lines. The binding orientation of zonisamide as determined by X-ray crystallography is shown in cyan [24].

The binding orientations of **4k** and **5k** are illustrated in Figure 6 and show that the inhibitors exhibited similar binding modes to zonisamide with respect to the placement of the sulfonamide group in proximity to the FAD cofactor. For **4k**, the amine group of the sulfonamide projected to the back of the cavity, enabling the formation of hydrogen bond interactions with Tyr398 and a water molecule, while the sulfonamide oxygen was also hydrogen-bonded to a water molecule. Other key interactions included pi–pi stacking between the benzenesulfonamide ring and Tyr398 and between the oxadiazole ring and Tyr326. A pi–sigma interaction was observed between the chlorophenyl and Ile199, while pi–sulfur interactions occurred between the sulfonamide group and Tyr60 as well as the FAD and between Cys172 and the oxadiazole moiety. As mentioned in the Introduction, in the reported structure of zonisamide complexed with MAO-B, the interactions that were highlighted included hydrogen bonding with the side chain of Gln206 as well as hydrogen bonding with two conserved water molecules, of which one forms a bridge between Lys296 and the N(5) of the FAD [24]. For safinamide, the X-ray crystal structure shows that the amide is also involved in hydrogen bonding with Gln206 and an ordered water molecule, while the fluorobenzyloxy side chain projects into the entrance cavity where it provides inhibitor stabilization [35].

For **5k**, the amine group of the sulfonamide projected, like zonisamide, to the front of the cavity, which enabled hydrogen bonding to Gln206 and a water molecule. A sulfonamide oxygen was hydrogen-bonded to a water molecule, while Tyr326 formed a hydrogen bond interaction with the oxygen of the oxadiazole ring. Other interactions that were observed included the pi–pi stacking of all three aromatic rings of the inhibitor with Tyr326 as well as pi–pi stacking between the benzenesulfonamide ring and Tyr398. The dichlorophenyl underwent a pi–sigma interaction with Ile199, and pi–sulfur interactions also occurred between the sulfonamide group and Tyr60 as well as the FAD. These interactions highlighted the importance of residues Ile199, Gln206 Tyr326, Tyr398 and the FAD for inhibitor stabilization (see Supplementary Materials). It was also noteworthy that the inhibitors mimicked the binding mode of zonisamide with respect to the placement of the sulfonamide group, with the key difference being the exact placement of the amine group, which determined whether hydrogen bonding occurred with Tyr398 or Gln206. The position of the benzenesulfonamide and oxadiazole rings of **5k** differed from that of **4k**, which indicated that different binding modes of the two oxadiazole isomers were required for the optimal placement of the sulfonamide moiety in the MAO-B cavity. Significantly, this difference enabled the oxadiazole ring of **5k** to form a hydrogen bond with Tyr326. The calculated interaction energies (e.g., CDOCKER_INTERACTION_ENERGY) for the complexes of **4k** and **5k** were 12.4 and −2.4 kcal/mol, respectively, while the corresponding value for zonisamide was 29.3 kcal/mol. This agrees with the higher potency of the synthetic sulfonamides compared to zonisamide.

## 3. Experimental Section

### 3.1. Materials and Methods

Starting amidoximes were prepared from commercial nitriles according to the literature procedures [36,37,38]. Except for derivatives **4l**, **4w**, **4z**, **5f**, **5g**, **5h**, **5k** and **5l**, the preparation and characterization of compounds **4** and **5** were described in our previous publications [39,40]. All other reagents and solvents were purchased from Merck and were used without further purification. Reactions were monitored by analytical thin layer chromatography (TLC) with Macherey-Nagel TLC sheets (Polygram SL G/UV 254) (Macherey-nagel GmbH & Co. KG, Düren, Germany) using UV light at 254 nm or fluorescence quenching for detection. NMR spectra were recorded with a Bruker Avance DPX 400 (Bruker Optics GmbH, Ettlingen, Germany) instrument (376 MHz and 101 MHz for ^1^H and ^13^C, respectively) in DMSO-*d*_6_. Chemical shifts are reported as parts per million (*δ*, ppm); the solvent peaks were used as internal standards: 2.50 ppm for residual ^1^H and 39.52 ppm for ^13^C in DMSO–*d*_6_. Multiplicities are abbreviated as follows: s, singlet; d, doublet; t, triplet; q, quartet; m, multiplet; br, broad. Coupling constants, *J*, are reported in Hertz (Hz). Melting points were determined in open capillary tubes with an Electrothermal IA 9300 series digital melting point apparatus (Electrothermal Engineering Ltd., Stone, United Kingdom). High-resolution mass spectra (HRMS) were measured with Bruker Maxis-qTOF (Bruker Daltonics GmbH & Co. KG, Bremen, Germany) (ESI, negative ionization mode).

### 3.2. Synthetic Organic Chemistry

#### 3.2.1. General Procedure for Synthesis of 1,2,4-Oxadiazoles **4l**, **4w**, **4z**, **5f**, **5g**, **5h**, **5k** and **5l** via Reaction of Amidoximes and Carboxylic Acids

CDI (1.0 mmol) was added to a stirred suspension of a carboxylic acid (1 mmol) in dry DMSO (3–5 mL). The reaction mixture was stirred at room temperature for 1 h, an amidoxime (1 mmol) was added and stirring was continued for 18 h at room temperature. Then, powdered NaOH (1.2 mmol) was added rapidly to the reaction mixture, and the reaction was stirred at room temperature for 2 h. After the reaction completed, the reaction mixture was cooled to 25 °C, and a 5% aq. solution of NaHCO_3_ (30 mL) was added. The resulting precipitate was collected by filtration, washed with cold water (2 × 10 mL) and air-dried at 50 °C.

**4-(3-(4-Bromophenyl)-1,2,4-oxadiazol-5-yl)benzenesulfonamide (4l).** White powder; 86% yield; mp 251–252 °C. ^1^H NMR (400 MHz, DMSO-*d_6_*) *δ* 8.38 (d, *J* = 8.6, 2H), 7.99–8.13 (m, 4H), 7.84 (dd, *J* = 8.3, 2.0 Hz, 2H), 7.65 (s, 2H). ^13^C NMR (101 MHz, DMSO-*d_6_*) *δ* 175.3, 168.5, 148.6, 133.2, 129.8, 129.5, 127.5, 126.6, 126.1, 125.8. HRMS (ESI), *m*/*z*: [M+H]^+^ calcd for C_14_H_10_BrN_3_O_3_S 379.9699; found 379.9702.

**4-(3-(Pyridin-2-yl)-1,2,4-oxadiazol-5-yl)benzenesulfonamide (4w).** White powder; 70% yield; mp 250–251 °C. ^1^H NMR (400 MHz, DMSO-*d_6_*) *δ* 8.86 (s, 1H), 8.24–8.43 (m, 3H), 8.14 (s, 1H), 8.05 (d, *J* = 8.0 Hz, 2H), 7.75 (s, 1H), 7.55 (s, 2H). ^13^C NMR (101 MHz, DMSO-*d_6_*) *δ* 175.5, 168.2, 151.3, 147.4, 143.3, 138.9, 129.6, 128.5, 128.3, 127.4, 125.3. HRMS (ESI), *m*/*z*: [M+H]^+^ calcd for C_13_H_10_N_4_O_3_S 303.0547; found 303.0547.

**4-(3-Methyl-1,2,4-oxadiazol-5-yl)benzenesulfonamide (4z).** White powder; 69% yield; mp 203–204 °C. ^1^H NMR (400 MHz, DMSO-*d_6_*) *δ* 8.18 (d, *J* = 8.1 Hz, 2H), 8.00 (d, *J* = 8.1 Hz, 2H), 7.54 (s, 2H), 2.68 (s, 3H). ^13^C NMR (101 MHz, DMSO-*d_6_*) *δ* 178.6, 167.4, 147.1, 129.9, 128.2, 127.3, 12.7. HRMS (ESI), *m*/*z*: [M+H]^+^ calcd for C_9_H_9_N_3_O_3_S 240.0437; found 240.0438.

**4-(5-(4-Chlorophenyl)-1,2,4-oxadiazol-3-yl)benzenesulfonamide (5f).** White powder; 80% yield; mp > 260 °C. ^1^H NMR (400 MHz, DMSO-*d_6_*) 8.37 (d, *J* = 8.4 Hz, 2H), 7.97 (d, *J* = 8.4 Hz, 2H), 7.80 (d, *J* = 8.5 Hz, 2H), 7.57 (d, *J* = 8.8 Hz, 2H), 7.13 (s, 2H). ^13^C NMR (101 MHz, DMSO-*d_6_*) *δ* 163.3, 157.1, 148.5, 136.0, 132.8, 131.1, 130.9, 129.5, 129.2, 126.5. HRMS (ESI), *m*/*z*: [M+H]^+^ calcd for C_14_H_10_ClN_3_O_3_S 336.0204; found 336.0201.

**4-(5-Methyl-1,2,4-oxadiazol-3-yl)benzenesulfonamide (5g).** White powder; 85% yield; mp 214–215 °C. ^1^H NMR (400 MHz, DMSO-*d_6_*) δ 8.27 (d, *J* = 8.8 Hz, 2H), 8.03 (d, *J* = 8.7 Hz, 2H), 7.56 (s, 2H), 2.44 (s, 3H). ^13^C NMR (101 MHz, DMSO-*d_6_*) δ 174.4, 168.6, 148.6, 129.2, 127.4, 126.7, 11.9. HRMS (ESI), *m*/*z*: [M+H]^+^ calcd for C_9_H_9_N_3_O_3_S 240.0438; found 240.0438.

**4-(5-(4-Fluorophenyl)-1,2,4-oxadiazol-3-yl)benzenesulfonamide (5h).** White powder; 66% yield; mp 254–255 °C. ^1^H NMR (400 MHz, DMSO-*d_6_*) *δ* 8.22–8.32 (m, 4H), 8.04 (d, *J* = 8.2 Hz, 2H), 7.58 (s, 2H), 7.52 (t, *J* = 8.8 Hz, 2H).^13^C NMR (101 MHz, DMSO-*d_6_*) *δ* 175.66, 168.12, 165.63 (d, ^1^*J_C-F_* = 251 Hz) 147.36, 131.61 (d, ^3^*J_C-F_* = 9 Hz), 129.67, 128.46, 127.33, 120.63, 117.61 (d, ^2^*J_C-F_* = 22 Hz). HRMS (ESI), *m*/*z*: [M+H]^+^ calcd for C_14_H_10_FN_3_O_3_S 320.0500; found 320.0504.

**4-(5-(3,4-Dichlorophenyl)-1,2,4-oxadiazol-3-yl)benzenesulfonamide (5k).** White powder; 55% yield; mp 208–210 °C. ^1^H NMR (400 MHz, DMSO-*d_6_*) *δ* 8.38 (d, *J* = 1.6 Hz, 1H), 8.28 (d, *J* = 8.3 Hz, 2H), 8.15 (dd, *J* = 8.4, 1.7 Hz, 1H), 8.05 (d, *J* = 8.3 Hz, 2H), 7.94 (d, *J* = 8.4 Hz, 1H), 7.52 (s, 2H). ^13^C NMR (101 MHz, DMSO) *δ* 174.8, 168.5, 147.7, 137.2, 133.4, 132.6, 130.3, 129.6, 128.7, 128.5, 127.3. HRMS (ESI), *m*/*z*: [M+H]^+^ calcd for C_14_H_9_Cl_2_N_3_O_3_S 369.9815; found 369.9817.

**4-(5-(Trifluoromethyl)-1,2,4-oxadiazol-3-yl)benzenesulfonamide (5l).** White powder; 82% yield; mp 195–196 °C. ^1^H NMR (400 MHz, DMSO-*d_6_*) δ 8.27 (dd, *J* = 8.2, 2.0 Hz, 2H), 8.05 (dd, *J* = 8.2, 1.9 Hz, 2H), 7.61 (s, 2H). ^13^C NMR (101 MHz, DMSO-*d_6_*) δ 168.3, 166.0 (q, ^2^*J_C-F_* = 44 Hz), 165.78, 148.1, 128.8, 128.0, 127.5, 116.4 (q, ^1^*J_C-F_* = 275 Hz). HRMS (ESI), *m*/*z*: [M+H]^+^ calcd for C_9_H_6_F_3_N_3_O_3_S 294.0155; found 294.0156.

#### 3.2.2. General Procedure for Synthesis of 1,2,4-Oxadiazoles **6a**–**g**

Cyclic anhydride **10** (2.5 mmol) was added to a solution of amidoxime **9** (2.5 mmol) in DMSO (2–3 mL). The reaction mixture was stirred at room temperature for 2–24 h. Subsequently, powdered NaOH (5 mmol) was rapidly added, and the reaction mixture was stirred at room temperature for 2 h. The reaction mixture was then diluted with cold water (30 mL) followed by the addition of hydrochloric acid to pH~1. The resulting precipitate was collected by filtration, washed with cold water (25 mL) and air-dried at 50 °C.

**4-(3-(4-Sulfamoylphenyl)-1,2,4-oxadiazol-5-yl)butanoic acid (6a).** Pale beige powder; 70% yield; mp 192–193 °C. ^1^H NMR (400 MHz, DMSO-*d*_6_) *δ* 12.21 (s, 1H), 8.19 (d, *J* = 8.3 Hz, 2H), 8.00 (d, *J* = 8.4 Hz, 2H), 7.55 (s, 2H), 3.06 (t, *J* = 7.5 Hz, 2H), 2.41 (t, *J* = 7.2 Hz, 2H), 2.02 (p, *J* = 7.3 Hz, 2H). ^13^C NMR (101 MHz, DMSO-*d*_6_) *δ* 181.09, 174.48, 167.34, 147.16, 129.90, 128.33, 127.27, 33.17, 25.80, 22.01. HRMS (ESI), *m*/*z*: [M+H]^+^ calcd for C_12_H_13_N_3_O_5_S 312.0649; found 312.0648.

**4-(3-(3-Sulfamoylphenyl)-1,2,4-oxadiazol-5-yl)butanoic acid (6b).** White powder; 69% yield; mp 123–124 °C. ^1^H NMR (400 MHz, DMSO-*d*_6_) *δ* 12.21 (s, 1H), 8.45 (s, 1H), 8.21 (d, *J* = 7.7 Hz, 1H), 8.02 (d, *J* = 7.8 Hz, 1H), 7.77 (t, *J* = 7.8 Hz, 1H), 7.56 (s, 2H), 3.07 (t, *J* = 7.5 Hz, 2H), 2.41 (t, *J* = 7.3 Hz, 2H), 2.03 (p, *J* = 7.3 Hz, 2H). ^13^C NMR (101 MHz, DMSO-*d*_6_) *δ* 181.14, 174.47, 167.34, 145.82, 130.94, 130.69, 129.09, 127.68, 124.83, 33.16, 25.80, 22.03. HRMS (ESI), *m*/*z*: [M+H]^+^ calcd for C_12_H_13_N_3_O_5_S 312.0649; found 312.0648.

**3-(3-(4-Sulfamoylphenyl)-1,2,4-oxadiazol-5-yl)propanoic acid (6c).** Pale beige powder; 70% yield; mp 195–196 °C. ^1^H NMR (400 MHz, DMSO-*d_6_*) *δ* 12.47 (s, 1H), 8.18 (d, *J* = 8.0 Hz, 2H), 8.00 (d, *J* = 8.1 Hz, 2H), 7.55 (s, 2H), 3.22 (t, *J* = 6.9 Hz, 2H), 2.86 (t, *J* = 6.9 Hz, 2H). ^13^C NMR (101 MHz, DMSO-*d_6_*) *δ* 180.9, 173.4, 167.3, 147.2, 129.8, 128.3, 127.3, 30.5, 22.4 HRMS (ESI), *m*/*z*: [M+H]^+^ calcd for C_11_H_11_N_3_O_5_S 298.0492; found 298.0492.

**3-(3-(3-Sulfamoylphenyl)-1,2,4-oxadiazol-5-yl)propanoic acid (6d).** White powder; 57% yield; mp 158–159 °C. ^1^H NMR (400 MHz, DMSO-*d_6_*) *δ* 12.48 (s, 1H), 8.44 (s, 1H), 8.16–8.26 (m, 1H), 8.02 (d, *J* = 8.3 Hz, 1H), 7.78 (t, *J* = 7.8 Hz, 1H), 7.56 (s, 2H), 3.22 (t, *J* = 6.9 Hz, 2H), 2.87 (t, *J* = 6.9 Hz, 2H). ^13^C NMR (101 MHz, DMSO-*d_6_*) *δ* 180.9, 173.4, 167.3, 145.8, 130.9, 130.7, 129.1, 127.6, 124.8, 30.5, 22.4. HRMS (ESI), *m*/*z*: [M+H]^+^ calcd for C_11_H_11_N_3_O_5_S 298.0492; found 298.0494.

**2-(3-(4-Sulfamoylphenyl)-1,2,4-oxadiazol-5-yl)benzoic acid (6e).** White powder; 85% yield; mp 215–216 °C. ^1^H NMR (400 MHz, DMSO-*d_6_*) *δ* 13.57 (s, 1H), 8.28 (d, *J* = 8.4 Hz, 2H), 8.04 (d, *J* = 8.4 Hz, 2H), 7.89–8.03 (m, 2H), 7.83 (dd, *J* = 6.5, 2.9 Hz, 2H), 7.58 (s, 2H). ^13^C NMR (101 MHz, DMSO-*d_6_*) *δ* 177.2, 167.9, 167.7, 147.39, 133.5, 133.3, 132.7, 131.2, 130.6, 129.7, 128.5, 127.4, 124.3. HRMS (ESI), *m*/*z*: [M+H]^+^ calcd for C_15_H_11_N_3_O_5_S 346.0492; found 346.0492.

**2-(3-(3-Sulfamoylphenyl)-1,2,4-oxadiazol-5-yl)benzoic acid (6f).** White powder; 84% yield; mp 219–220 °C. ^1^H NMR (400 MHz, DMSO-*d_6_*) *δ* 13.58 (s, 1H), 8.53 (s, 1H), 8.30 (d, *J* = 8.1 Hz, 1H), 8.06 (d, *J* = 8.1 Hz, 1H), 7.98–8.04 (m, 1H), 7.91–7.98 (m, 1H), 7.83 (s, 3H), 7.58 (s, 2H). ^13^C NMR (101 MHz, DMSO-*d_6_*) *δ* 177.2, 167.9, 167.7, 145.9, 133.4, 133.3, 132.7, 131.2, 131.1, 130.8, 130.6, 129.3, 127.5, 124.9, 124.4. HRMS (ESI), *m*/*z*: [M+H]^+^ calcd for C_15_H_11_N_3_O_5_S 346.0492; found 346.0491.

**2-(3-(4-Sulfamoylphenyl)-1,2,4-oxadiazol-5-yl)cyclohexane-1-carboxylic acid (6g).** White powder; 77% yield; mp 170–171 °C. ^1^H NMR (400 MHz, DMSO-*d_6_*) *δ* 12.37 (s, 1H), 8.17 (d, *J* = 8.4 Hz, 2H), 8.00 (d, *J* = 8.4 Hz, 2H), 7.55 (s, 2H), 3.56 (d, *J* = 5.0 Hz, 1H), 2.51 (s, 1H), 2.05 (s, 3H), 1.84 (s, 1H), 1.60 (s, 1H), 1.48 (s, 3H). ^13^C NMR (101 MHz, DMSO-*d_6_*) *δ* 182.9, 174.7, 167.0, 147.2, 129.9, 128.3, 127.3, 43.2, 36.1, 26.9, 26.5, 23.7, 23.4. HRMS (ESI), *m*/*z*: [M+H]^+^ calcd for C_15_H_17_N_3_O_5_S 352.0962; found 352.0692.

### 3.3. MAO Inhibition Studies

The inhibitory activities of the synthesized compounds were evaluated with commercially available recombinant human MAO-A and MAO-B (Merck), following a previously reported protocol [41,42]. Kynuramine served as the substrate for both MAO isoforms and was oxidized to yield 4-hydroxyquinoline, a fluorescent metabolite. The levels of 4-hydroxyquinoline produced in the reactions were determined using fluorescence spectrometry (λ_ex_ = 310 nm; λ_em_ = 400 nm). Using this method, kynuramine oxidation catalyzed by MAO was determined in the presence of a range of inhibitor concentrations (0.003–100 µM) and in the absence of an inhibitor. The resulting data were used to generate sigmoidal plots of the reaction rate versus the logarithm of inhibitor concentration (log[I]), from which IC_50_ values were derived and presented as the mean ± standard deviation (SD) of three measurements.

To determine the inhibition mechanism, Lineweaver–Burk plots were generated [41]. Six plots in total were prepared for the test inhibitor: five using inhibitor concentrations that spanned the IC_50_ value and one without an inhibitor. Each line was derived from measurements at eight kynuramine concentrations (15–250 µM). The slopes obtained from the Lineweaver–Burk plots were then graphed against inhibitor concentration, and the K_i_ value was calculated from the x-intercept of this secondary plot (K_i_ = –x-intercept).

Time-dependent inhibition was assessed by pre-incubating MAO-B with the test inhibitor at twice its experimentally determined IC_50_ for 0, 15, 30, or 60 min. The reactions were then started by adding an equal volume of kynuramine, resulting in a final inhibitor concentration equal to its IC_50_. Incubations were continued for an additional 20 min at room temperature, after which the production of 4-hydroxyquinoline (reflecting the remaining MAO catalytic activity) was quantified as previously described [41]. Parallel control assays were performed using the irreversible inhibitor, (R)-deprenyl.

### 3.4. Molecular Docking Studies

Molecular docking was performed following the methodology described in the literature [29,30]. All simulations were conducted using the Discovery Studio 3.1 life sciences software package (Accelrys, Inc., San Diego, CA, USA), employing the X-ray crystal structure of MAO-B complexed with zonisamide (PDB ID: 3PO7) as the protein model.

The docking workflow was designed to predict how the derivatives orient within the active site of MAO-B. The crystal structure was obtained from the Protein Data Bank, and ionizable residues were protonated according to predicted pKa values at physiological pH (7.4). Hydrogen atoms were added to the protein models, and the FAD cofactor was kept in its oxidized form. The geometries and valencies of the FAD and the co-crystallized ligand were checked to ensure correctness. Atomic interactions were described using the CHARMm force field (Momany and Rone implementation), and the protein backbone was restrained with a fixed-atom constraint. After resolving steric clashes, the system was energy-minimized with the Smart Minimizer algorithm (up to 50,000 steps) using the generalized Born implicit solvent model. Binding site preparation involved removing the co-crystallized ligand and non-essential water molecules, while conserved waters (MAO-B: HOH1155, HOH1170, HOH1351) were retained. Ligands were built in Discovery Studio and geometry-optimized with a Dreiding-like force field (5000 iterations). The Prepare Ligands procedure was used to prepare the ligands, and they were subsequently typed with CHARMm to assign partial charges and atom-type potentials. Docking was carried out with CDOCKER, and for each compound, ten random starting conformations were produced, using a heating target of 700 K in full potential mode. The resulting poses were subsequently refined by in situ ligand minimization (Smart Minimizer) to improve binding interactions.

## 4. Conclusions

Based on our previous works that investigated the MAO inhibition properties of benzenesulfonamide derivatives, the present study applied a ‘scaffold hopping’ approach to design a series of 1,2,4-oxadiazole derivatives. These derivatives were primarily based on a series of 1,3,4-oxadiazole substituted benzenesulfonamide derivatives that possessed the highest MAO-B inhibition potencies among the reported compounds. This study discovered numerous compounds with submicromolar MAO-B inhibition potencies, with seven compounds exhibiting an IC_50_ < 0.02 µM (**4b**, **4c**, **4k**, **4l**, **4o**, **5h** and **5k**). The most potent MAO-B inhibitors were derivatives **4k** and **5k** with IC_50_ values of 0.0079 and 0.0045 µM, respectively. These compounds were significantly more potent than the reference MAO-B inhibitor, safinamide (IC_50_ = 0.176 µM). With a representative inhibitor (**4b**), it was shown that MAO-B inhibition was competitive and reversible. The derivatives were, for the most part, selective for the MAO-B isoform, compared to MAO-A, which agrees with our previous works on benzenesulfonamide derivatives. The most potent MAO-A inhibition was observed for **5c** and **5d** with IC_50_ values of 6.04 and 6.99 µM, respectively. This study concludes that 1,2,4-oxadiazole sulfonamide derivatives are a class of compounds with potent and selective MAO-B inhibition, with potencies comparable to the previously reported 1,3,4-oxadiazole substituted benzenesulfonamide derivatives (e.g., **3**). MAO-B-selective inhibitors are of interest to bioorganic and medicinal chemistry due to the role that MAO plays in several disease states. Areas where MAO-B inhibitors find application are in the treatment of Parkinson’s disease and potentially also in Alzheimer’s disease. There is thus a continued interest in MAO-B inhibitors as therapeutic agents, with several patents registered for these compounds and one compound undergoing clinical trials.

## Figures and Tables

**Table 1:** Inhibitory potencies of 1,2,4-oxadiazole sulfonamide derivatives **4a**–**ak**, **5a**–**l** and **6a**–**g**, as well as reference inhibitors, against human MAO ^a^.

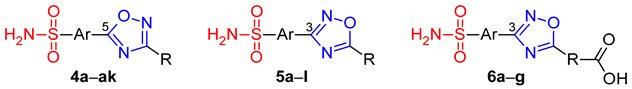				
Compound	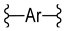	R	IC_50_, µM	
			MAO-A	MAO-B
**4a**	*p*-phenylene	Ph	82.9 ± 6.09	0.097 ± 0.0061
**4b**	*p*-phenylene	4-MeC_6_H_4_	NI ^b^	0.016 ± 0.0063
**4c**	*p*-phenylene	3-MeC_6_H_4_	NI ^b^	0.012 ± 0.0026
**4d**	*p*-phenylene	4-MeOC_6_H_4_	NI ^b^	0.149 ± 0.0074
**4e**	*p*-phenylene	2-MeOC_6_H_4_	NI ^b^	0.462 ± 0.015
**4f**	*p*-phenylene	3-MeOC_6_H_4_	NI ^b^	0.105 ± 0.0049
**4g**	*p*-phenylene	3,4-diMeOC_6_H_4_	NI ^b^	0.194 ± 0.0067
**4h**	*p*-phenylene	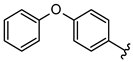	93.3 ± 17.0	0.086 ± 0.0052
**4i**	*p*-phenylene	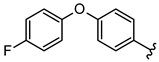	NI ^b^	0.287 ± 0.028
**4j**	*p*-phenylene	2-ClC_6_H_4_	15.9 ± 1.94	0.098 ± 0.0085
**4k**	*p*-phenylene	3-ClC_6_H_4_	95.2 ± 2.03	0.0079 ± 0.0015
**4l**	*p*-phenylene	4-BrC_6_H_4_	NI ^b^	0.011 ± 0.0015
**4m**	*p*-phenylene	4-FC_6_H_4_	NI ^b^	0.025 ± 0.00063
**4n**	*p*-phenylene	3-FC_6_H_4_	NI ^b^	0.190 ± 0.019
**4o**	*p*-phenylene	2-FC_6_H_4_	NI ^b^	0.014 ± 0.00021
**4p**	*p*-phenylene	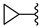	NI ^b^	NI ^b^
**4q**	*p*-phenylene	i-Propyl	NI ^b^	17.3 ± 1.34
**4r**	*p*-phenylene	4-t-BuC_6_H_4_	NI ^b^	0.143 ± 0.015
**4s**	*p*-phenylene	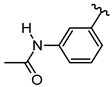	67.3 ± 20.7	0.058 ± 0.0040
**4t**	*p*-phenylene	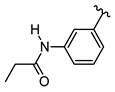	NI ^b^	0.859 ± 0.068
**4u**	*p*-phenylene	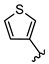	NI ^b^	0.364 ± 0.052
**4v**	*p*-phenylene	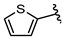	NI ^b^	0.309 ± 0.031
**4w**	*p*-phenylene	2-Pyridyl	NI ^b^	0.699 ± 0.079
**4x**	*p*-phenylene	3-Pyridyl	NI ^b^	5.59 ± 0.045
**4y**	*p*-phenylene	4-Pyridyl	84.3 ± 26.0	0.314 ± 0.0056
**4z**	*p*-phenylene	Me	NI ^b^	NI ^b^
**4aa**	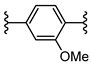	3-FC_6_H_4_	NI ^b^	0.093 ± 0.0023
**4ab**	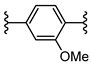	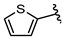	NI ^b^	0.706 ± 0.031
**4ac**	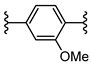	2-ClC_6_H_4_	84.4 ± 29.0	0.266 ± 0.016
**4ad**	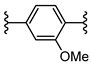	Ph	NI ^b^	0.414 ± 0.017
**4ae**	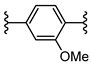	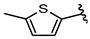	NI ^b^	0.368 ± 0.022
**4af**	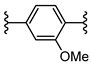	4-MeOC_6_H_4_	43.5 ± 11.2	0.166 ± 0.0035
**4ag**	*m*-phenylene	3-Pyridyl	67.0 ± 10.1	1.14 ± 0.059
**4ah**	*m*-phenylene	2-ClC_6_H_4_	24.1 ± 4.00	0.108 ± 0.0091
**4ai**	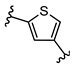	2-MeOC_6_H_4_	NI ^b^	4.45 ± 0.508
**4aj**	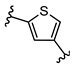	2-pyridyl	NI ^b^	NI ^b^
**4ak**	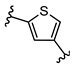	3-MeOC_6_H_4_	NI ^b^	0.159 ± 0.013
**5a**	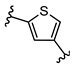	Ph	NI ^b^	0.183 ± 0.0071
**5b**	*m*-phenylene	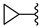	NI ^b^	NI ^b^
**5c**	*m*-phenylene	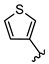	6.04 ± 0.048	0.517 ± 0.031
**5d**	*m*-phenylene	Ph	6.99 ± 0.318	0.095 ± 0.00091
**5e**	*p*-phenylene	Ph	19.7 ± 3.83	0.135 ± 0.010
**5f**	*p*-phenylene	4-ClC_6_H_4_	NI ^b^	0.649 ± 0.037
**5g**	*p*-phenylene	Me	NI ^b^	NI ^b^
**5h**	*p*-phenylene	4-FC_6_H_4_	NI ^b^	0.018 ± 0.0021
**5i**	*p*-phenylene	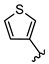	96.7 ± 17.0	0.339 ± 0.0022
**5j**	*p*-phenylene	4-CNC_6_H_4_	60.0 ± 20.8	0.136 ± 0.013
**5k**	*p*-phenylene	3,4-diClC_6_H_4_	66.4 ± 18.9	0.0045 ± 0.0015
**5l**	*p*-phenylene	CF_3_	NI ^b^	32.8 ± 10.6
**6a**	*p*-phenylene	-(CH_2_)_3_-	NI ^b^	NI ^b^
**6b**	*m*-phenylene	-(CH_2_)_3_-	NI ^b^	192 ± 2.26
**6c**	*p*-phenylene	-(CH_2_)_2_-	NI ^b^	NI ^b^
**6d**	*m*-phenylene	-(CH_2_)_2_-	NI ^b^	NI ^b^
**6e**	*p*-phenylene	*o*-phenylene	NI ^b^	160 ± 3.68
**6f**	*m*-phenylene	*o*-phenylene	NI ^b^	77.4 ± 7.50
**6g**	*p*-phenylene	1,2-cyclohexyl	NI ^b^	NI ^b^
Methylene blue			0.030 ± 0.0045	
Safinamide				0.176 ± 0.036

^a^ IC_50_ values are presented as the mean (±standard deviation) of 3 technical replicates; ^b^ NI—No inhibition observed at a maximal concentration of 100 µM.

## Data Availability

Data is contained within the article and Supplementary Materials.

## Supplementary Materials

The following supporting information can be downloaded at https://www.mdpi.com/article/10.3390/molecules31183326/s1, containing copies of NMR spectra for derivatives **4l**, **4w**, **4z**, **5f**, **5g**, **5h**, **5k**, **5l** and **6a**–**g** and HPLC traces for **4b**, **4k**, **5k**. Figures S1–S3: Two-dimensional diagrams and interaction plots for the docking study with **4k**, **5k** are also provided.

## Abbreviations

The following abbreviations are used in this manuscript:

Aβ	Amyloid-beta
CDI	1,1′-Carbonyldiimidazole
DMSO	Dimethyl sulfoxide
ESI	Electrospray ionization
FAD	Flavin adenine dinucleotide
HRMS	High-resolution mass spectra
IC_50_	Half-maximal inhibitory concentration
MAO	Monoamine oxidase
Mp	Melting point
NI	No inhibition
NMR	Nuclear magnetic resonance
PDB	Protein Data Bank
qTOF	Quadrupole time-of-flight
SAR	Structure–activity relationship
SD	Standard deviation
TLC	Thin layer chromatography
UV	Ultraviolet
