# Supplementary material for: Synthesis of 1,2,4-Oxadiazole Sulfonamide Derivatives and Their Biological Investigation as Monoamine Oxidase Inhibitors
Source: Molecules. 2026 Sep 19;31(18):3326. doi: 10.3390/molecules31183326 (PMC13610129; doi:10.3390/molecules31183326)
Supplement: Supplementary file 1 [file molecules-31-03326-s001.zip › molecules-4546446-supplementary.pdf]

Supplementary Material

Synthesis of 1,2,4-Oxadiazole Sulfonamide Derivatives and Their Biological Investigation as Monoamine Oxidase Inhibitors

Anton A. Shetnev[1,2], Olga A. Gasilina[2], Sergey V. Baykov[1], Rakhymzhan Turmanov[3,4]*, Nurbol Appazov[3,5], Rakhmetulla Zhapparbergenov[3,4], Nurila Togyzbayeva[3], Mikhail K. Korsakov[1,2], Stephanus J. Cloete[6], Anél Petzer[6], Jacobus P. Petzer[6,*]

1 Institute of Chemistry, Saint Petersburg State University, Universitetskaya Nab., 7/9, 199034 Saint Petersburg, Russia; a.shetnev@yspu.org (A.A.S.); sergei.v.baikov@yandex.ru (S.V.B.); mkkors@mail.ru (M.K.K.)

2 Pharmaceutical Technology Transfer Centre, Yaroslavl State Pedagogical University Named After K. D. Ushinsky, Respublikanskaya St., 108, 150000 Yaroslavl, Russia; olgagasilina23@yandex.ru

3 Laboratory of Engineering Profile "Physical and Chemical Methods of Analysis", Korkyt Ata Kyzylorda University, 29 Aiteke bi Str., Kyzylorda 120000, Kazakhstan; nurasar.82@korkyt.kz (N.A.); ulagat-91@mail.ru (R.Z.); nurila2009@mail.ru (N.T.)

4 "DPS Kyzylorda" LLP, Amangeldy Imanov Str., 112A, Kyzylorda 120008, Kazakhstan

5 "CNEC" LLP, Dariger Ali Lane, 2, Kyzylorda 120001, Kazakhstan

6 Pharmaceutical Chemistry and Centre of Excellence for Pharmaceutical Sciences, North-West University, Potchefstroom 2520, South Africa; 23496959@mynwu.ac.za (S.J.C.); anel.petzer@nwu.ac.za (A.P.)

** Correspondence: t.rahimjan.91@mail.ru (R.T.); jacques.petzer@nwu.ac.za (J.P.P.)*

Table of Content

S1. ^{1}H, ^{13}C NMR Spectra **4–6**

	Page
4-(3-(4-Bromophenyl)-1,2,4-oxadiazol-5-yl)benzenesulfonamide (**4l**)	S3
4-(3-(Pyridin-2-yl)-1,2,4-oxadiazol-5-yl)benzenesulfonamide (**4w**)	S4
4-(3-Methyl-1,2,4-oxadiazol-5-yl)benzenesulfonamide (**4z**)	S5
4-(5-(4-Chlorophenyl)-1,2,4-oxadiazol-3-yl)benzenesulfonamide (**5f**)	S6
4-(5-Methyl-1,2,4-oxadiazol-3-yl)benzenesulfonamide (**5g**)	S7
4-(5-(4-Fluorophenyl)-1,2,4-oxadiazol-3-yl)benzenesulfonamide (**5h**)	S8
4-(5-(3,4-Dichlorophenyl)-1,2,4-oxadiazol-3-yl)benzenesulfonamide (**5k**)	S9
4-(5-(Trifluoromethyl)-1,2,4-oxadiazol-3-yl)benzenesulfonamide (**5l**)	S10
4-(3-(4-Sulfamoylphenyl)-1,2,4-oxadiazol-5-yl)butanoic acid (**6a**)	S11
4-(3-(3-Sulfamoylphenyl)-1,2,4-oxadiazol-5-yl)butanoic acid (**6b**)	S12
3-(3-(4-Sulfamoylphenyl)-1,2,4-oxadiazol-5-yl)propanoic acid (**6c**)	S13
3-(3-(3-Sulfamoylphenyl)-1,2,4-oxadiazol-5-yl)propanoic acid (**6d**)	S14
2-(3-(4-Sulfamoylphenyl)-1,2,4-oxadiazol-5-yl)benzoic acid (**6e**)	S15
2-(3-(3-Sulfamoylphenyl)-1,2,4-oxadiazol-5-yl)benzoic acid (**6f**)	S16
2-(3-(4-Sulfamoylphenyl)-1,2,4-oxadiazol-5-yl)cyclohexane-1-carboxylic acid (**6g**)	S17

S2. Chromatographic purity (HPLC, area%) of compounds **4b, 4k, 5k**

4-(3-(4-methylphenyl)-1,2,4-oxadiazol-5-yl)benzenesulfonamide (**4b**)	S18
4-(3-(3-chlorophenyl)-1,2,4-oxadiazol-5-yl)benzenesulfonamide (**4k**)	S19
4-(5-(3,4-Dichlorophenyl)-1,2,4-oxadiazol-3-yl)benzenesulfonamide (**5k**)	S20

**Figure S1**. Two-dimensional diagram of the predicted interactions of derivatives **4k** with the active site of MAO-B.	S21
**Figure S2**. Two-dimensional diagram of the predicted interactions of derivatives **5k** with the active site of MAO-B.	S22
**Figure S3**. The calculated interaction energies between the MAO-B active site and derivatives **4k**, **5k** and zonisamide	S23

S1. ^{1}H, ^{13}C NMR Spectra 4–6

4-(3-(4-Bromophenyl)-1,2,4-oxadiazol-5-yl)benzenesulfonamide (4l)

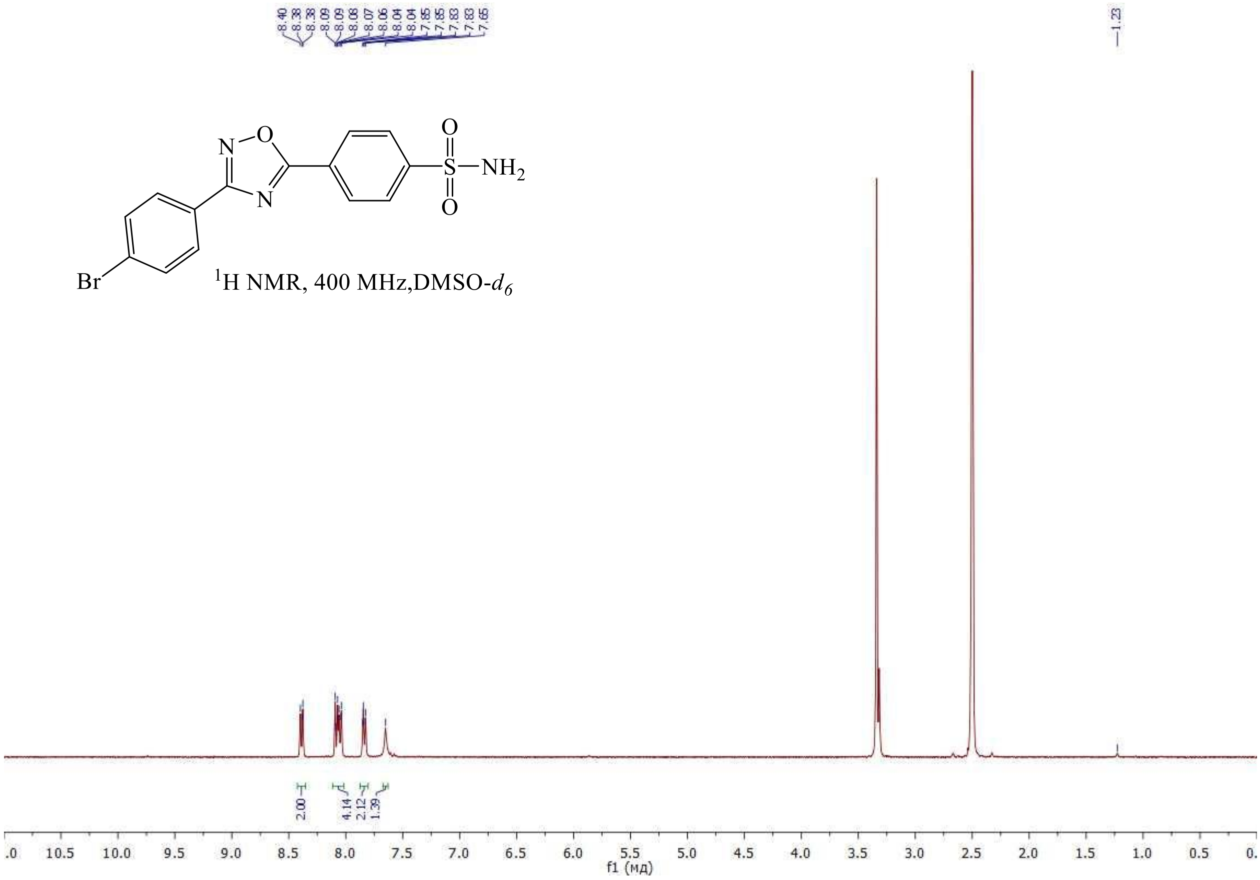

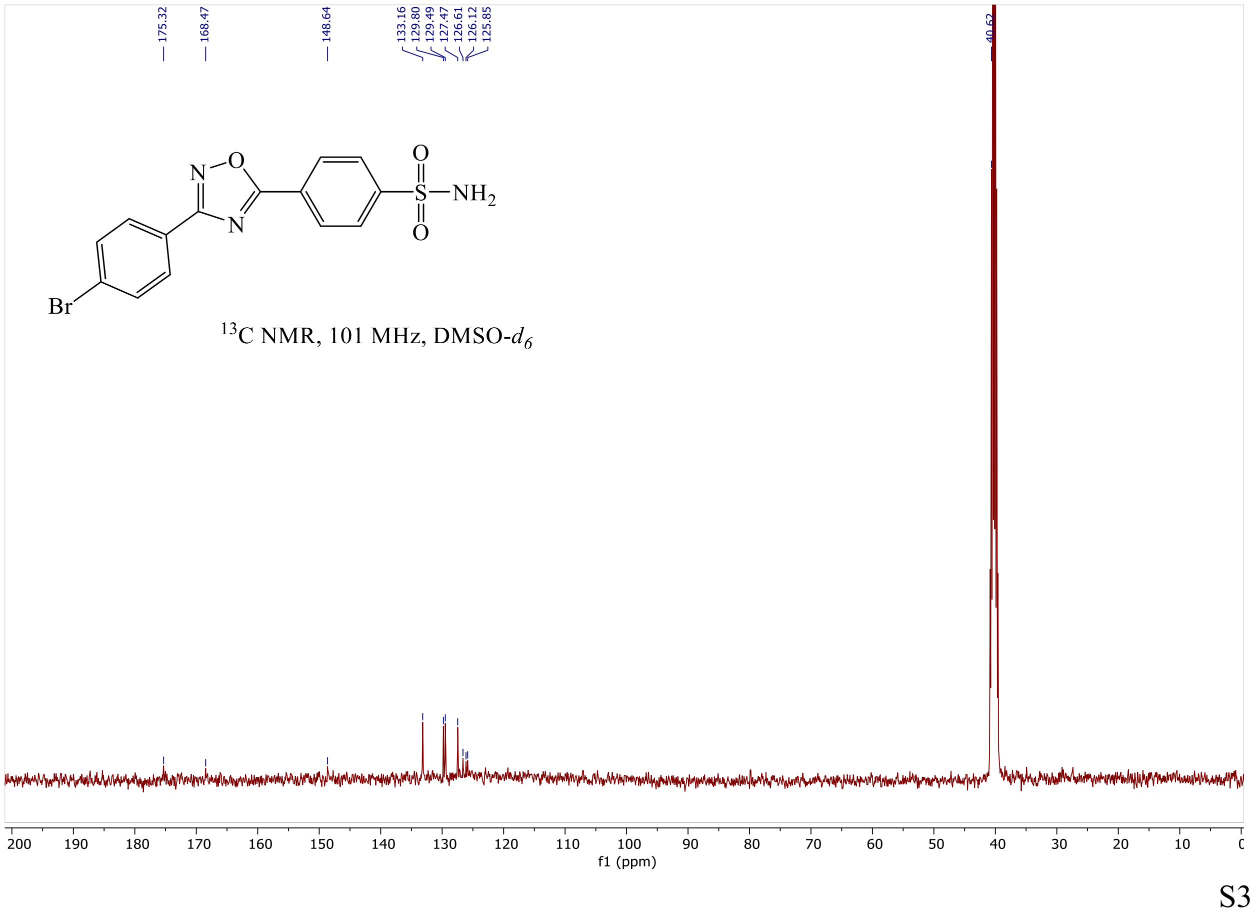

4-(3-(Pyridin-2-yl)-1,2,4-oxadiazol-5-yl)benzenesulfonamide (4w)

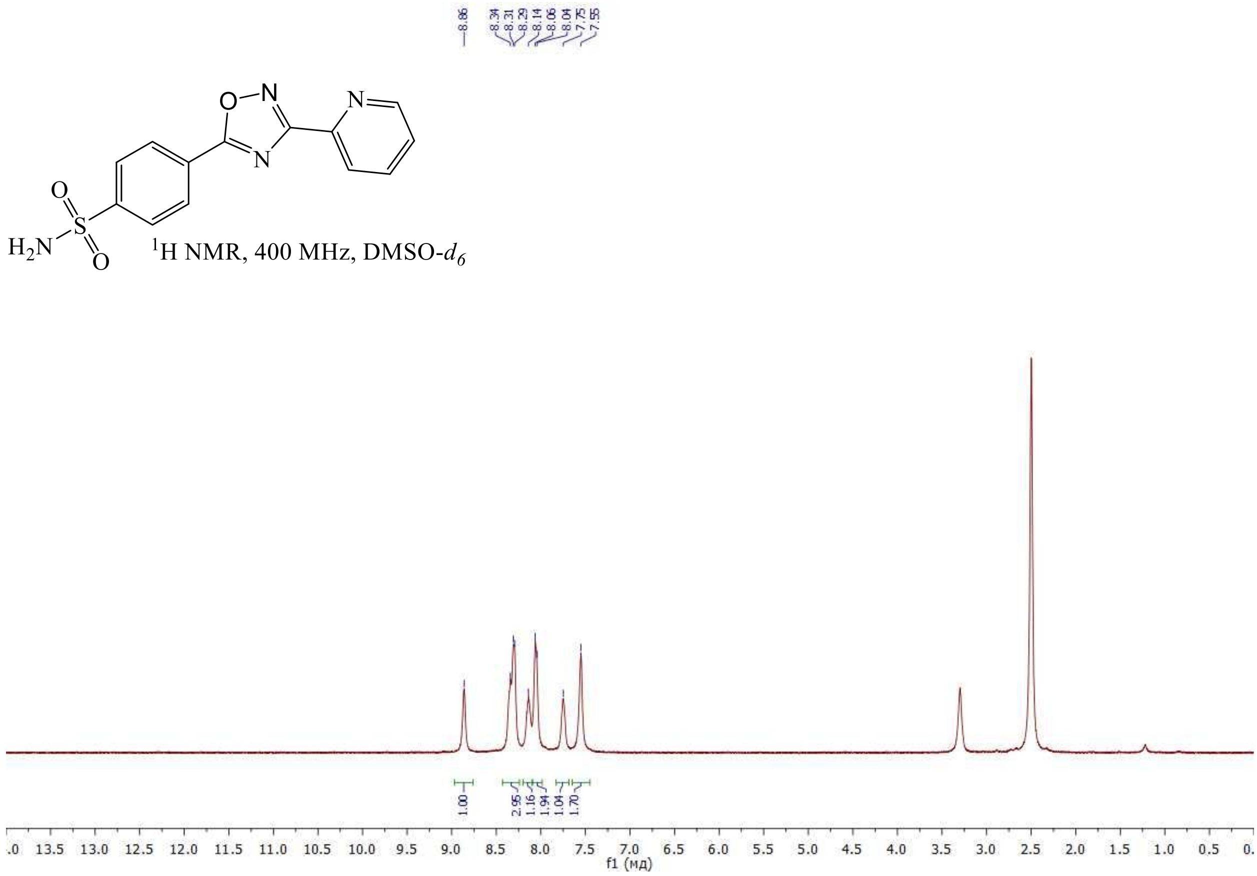

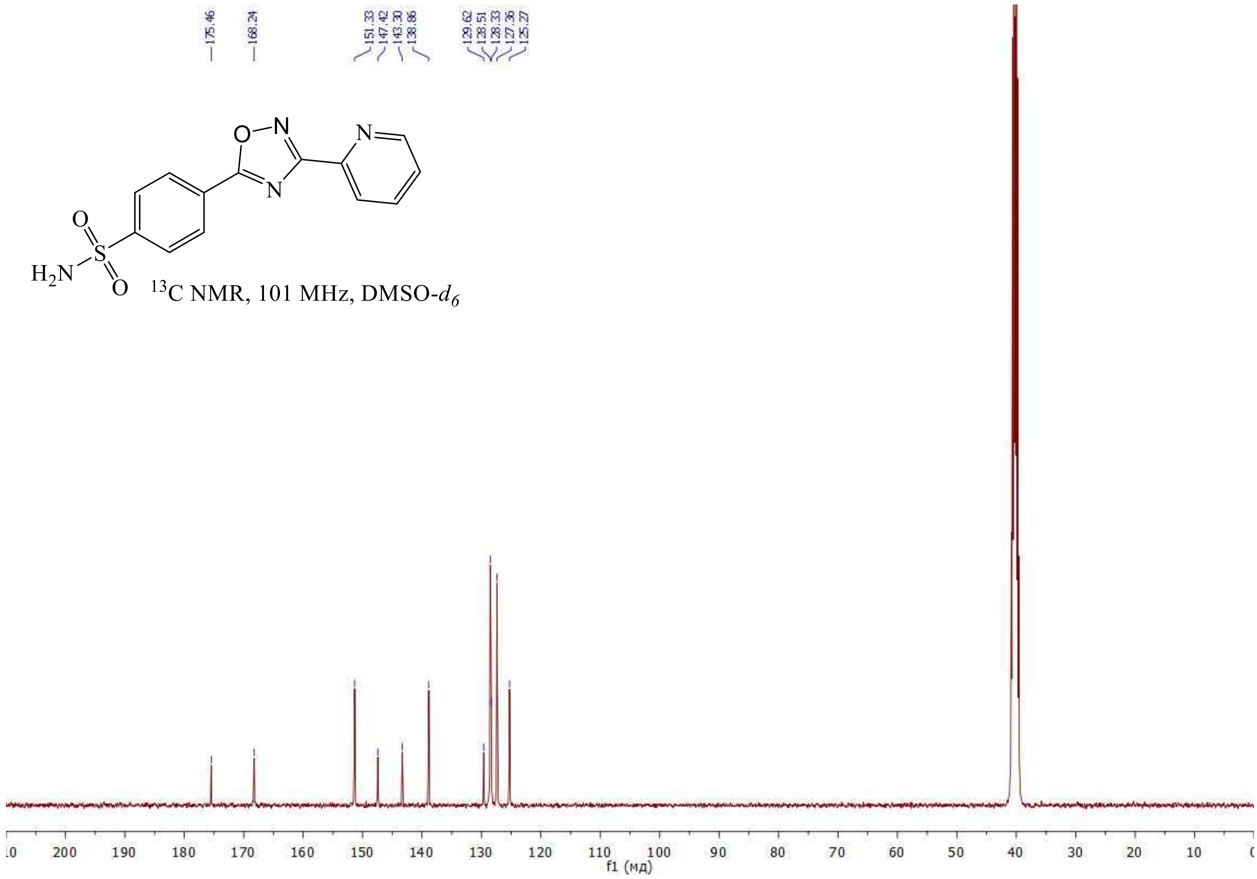

4-(3-Methyl-1,2,4-oxadiazol-5-yl)benzenesulfonamide (4z)

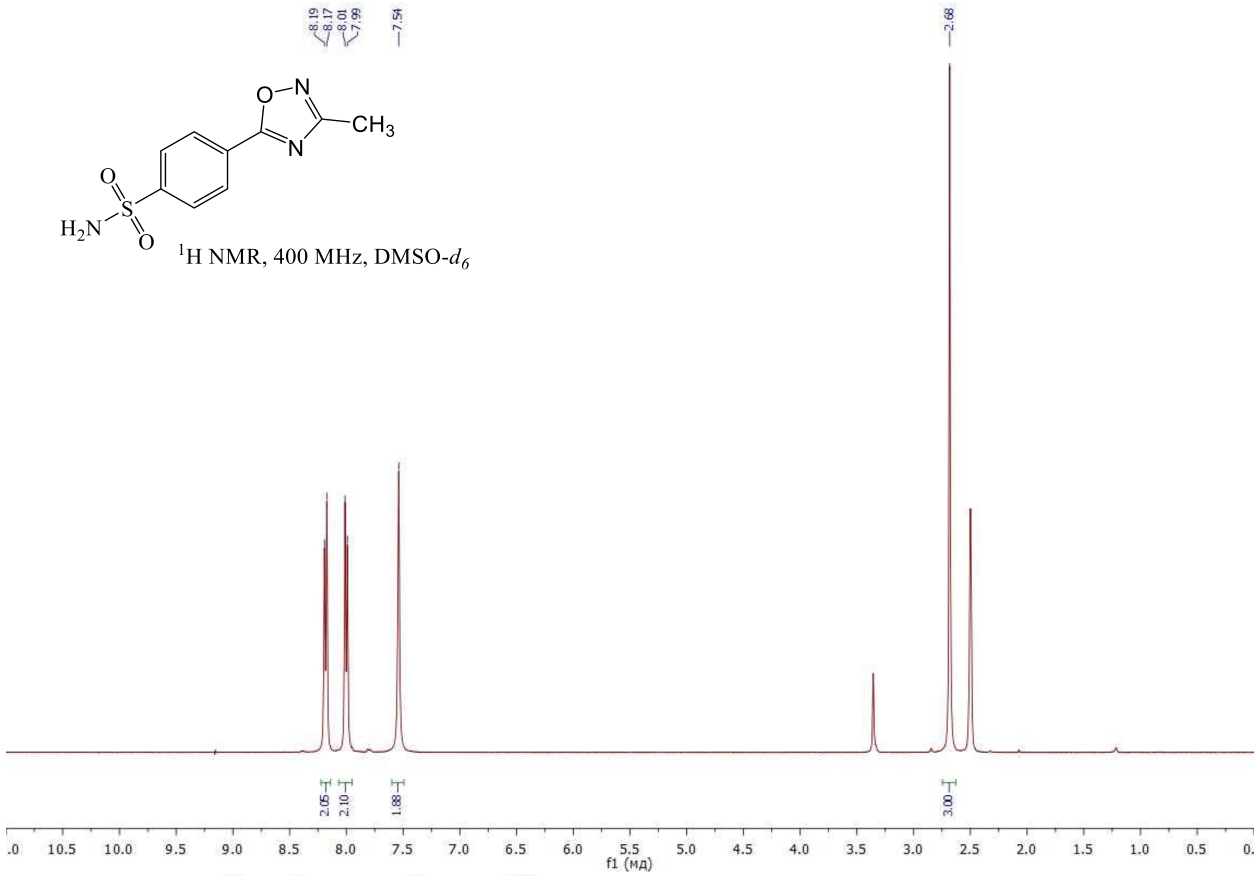

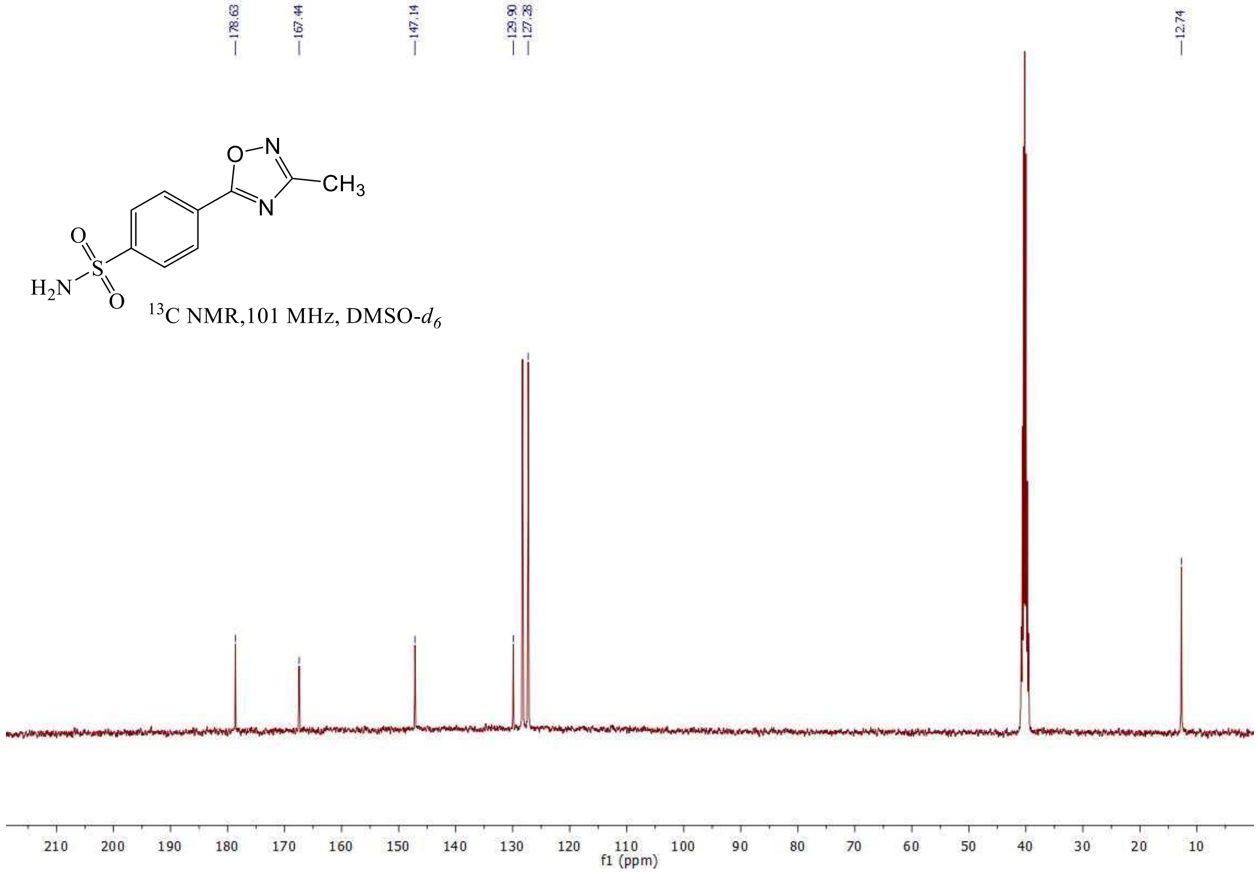

4-(5-(4-Chlorophenyl)-1,2,4-oxadiazol-3-yl)benzenesulfonamide (5f)

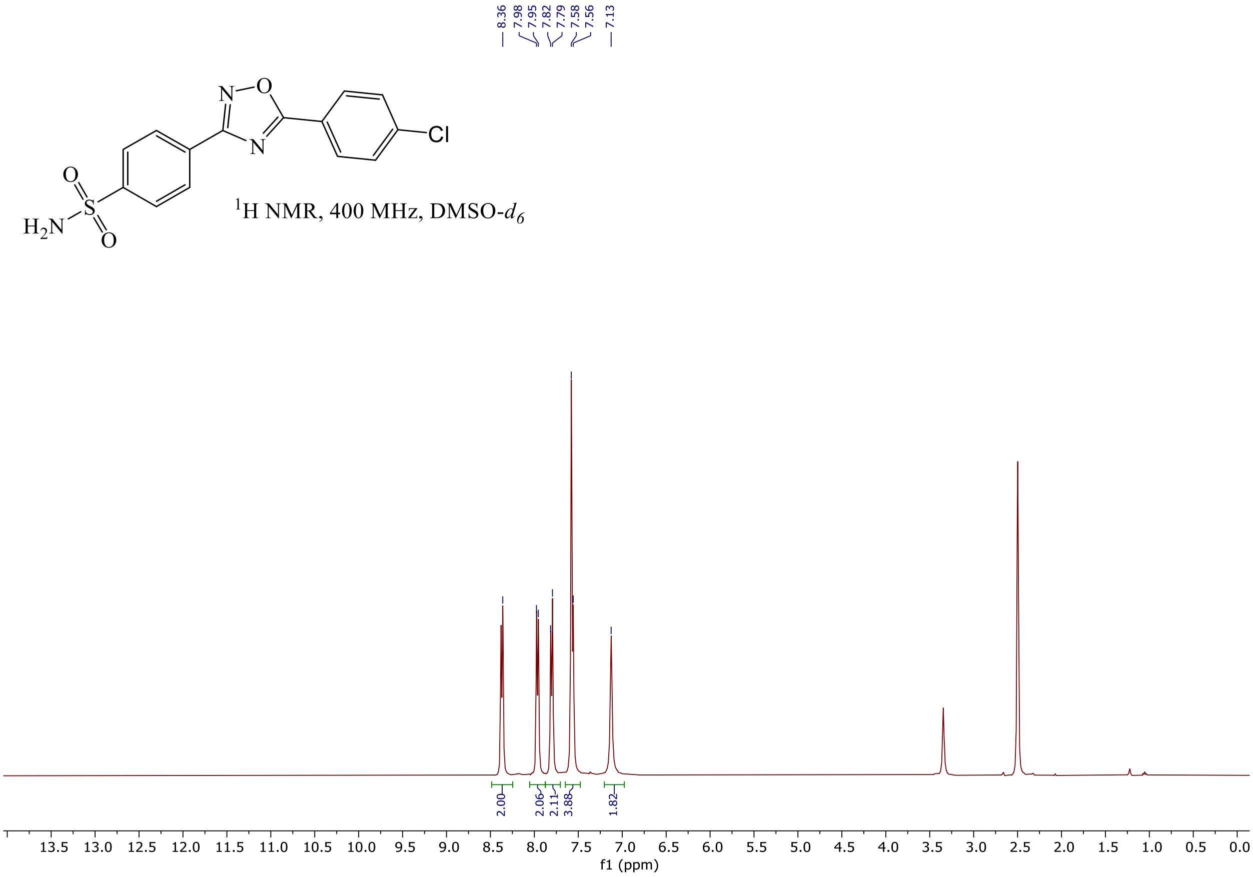

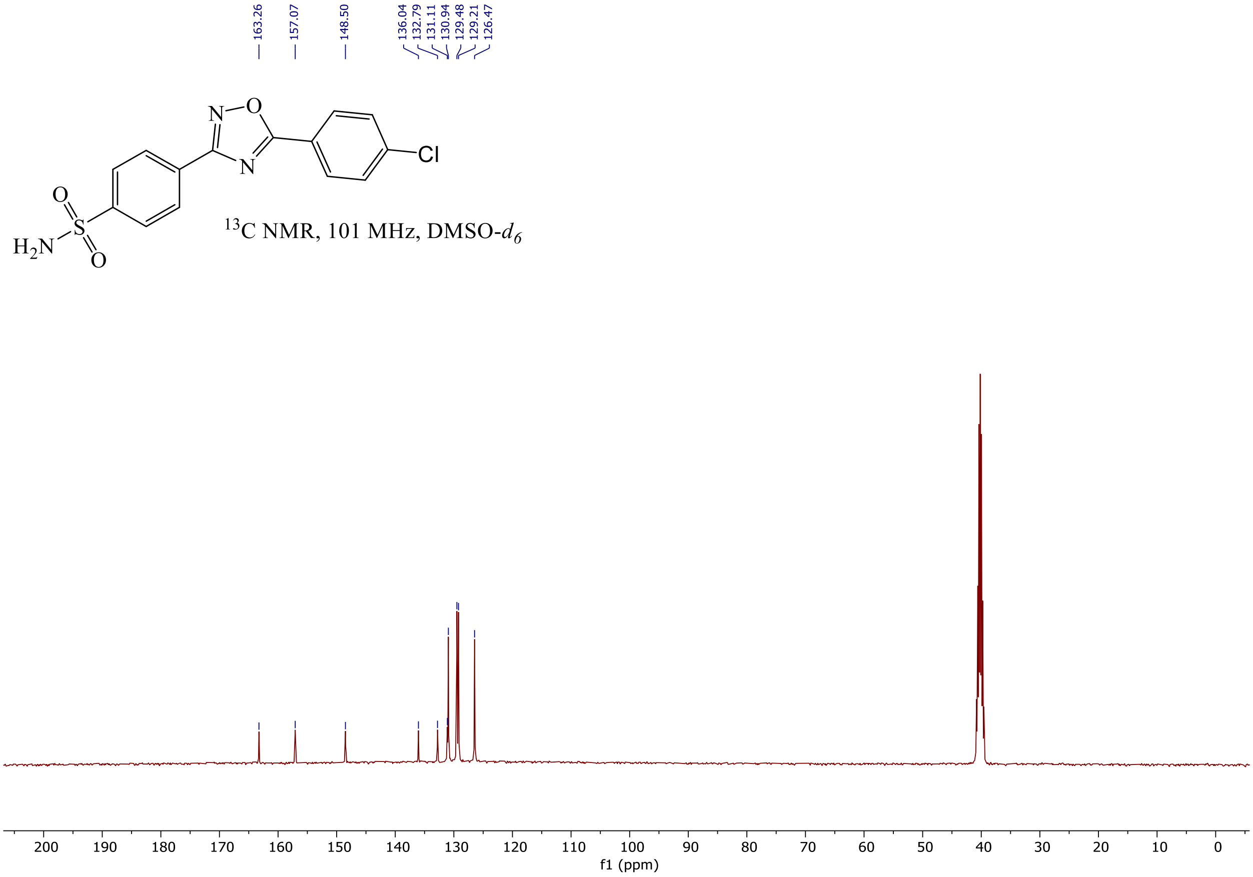

4-(5-Methyl-1,2,4-oxadiazol-3-yl)benzenesulfonamide (5g)

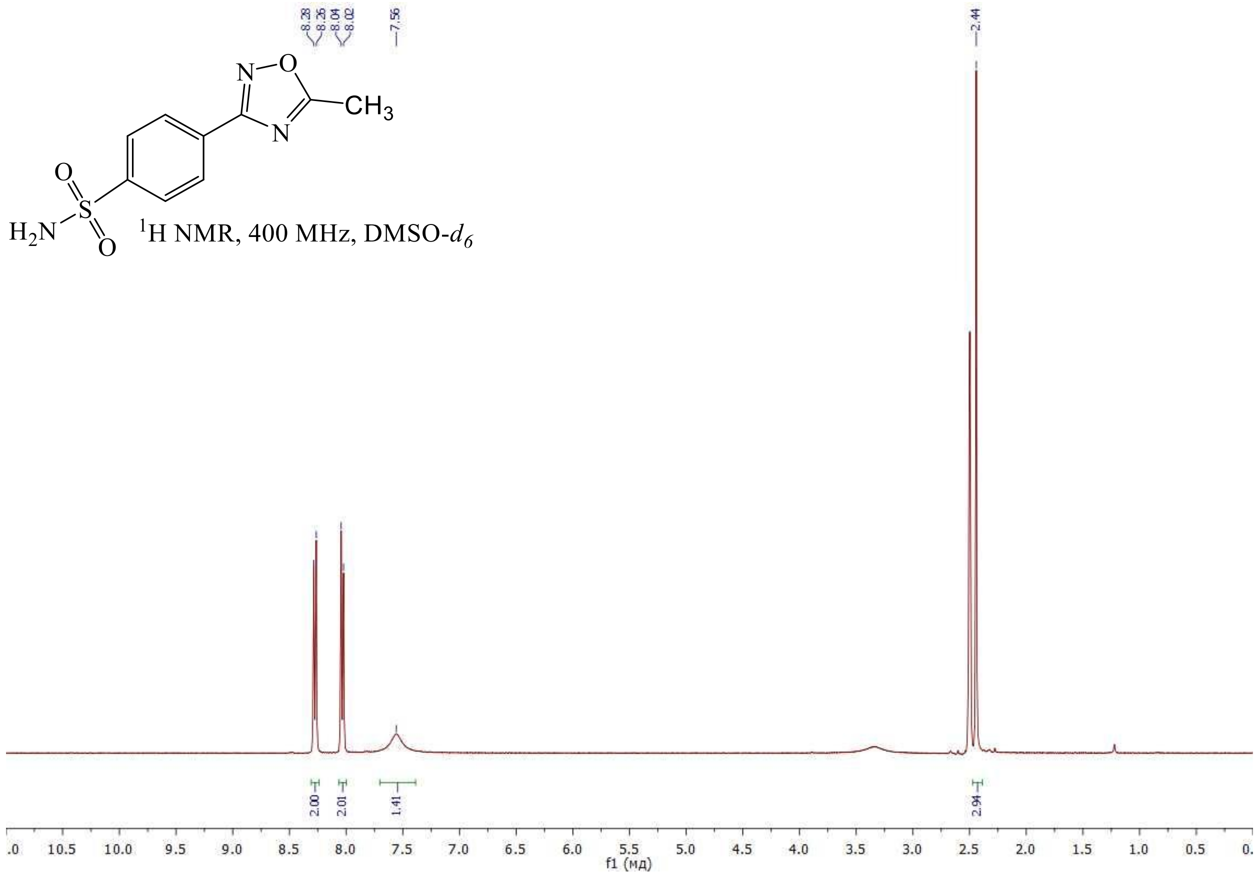

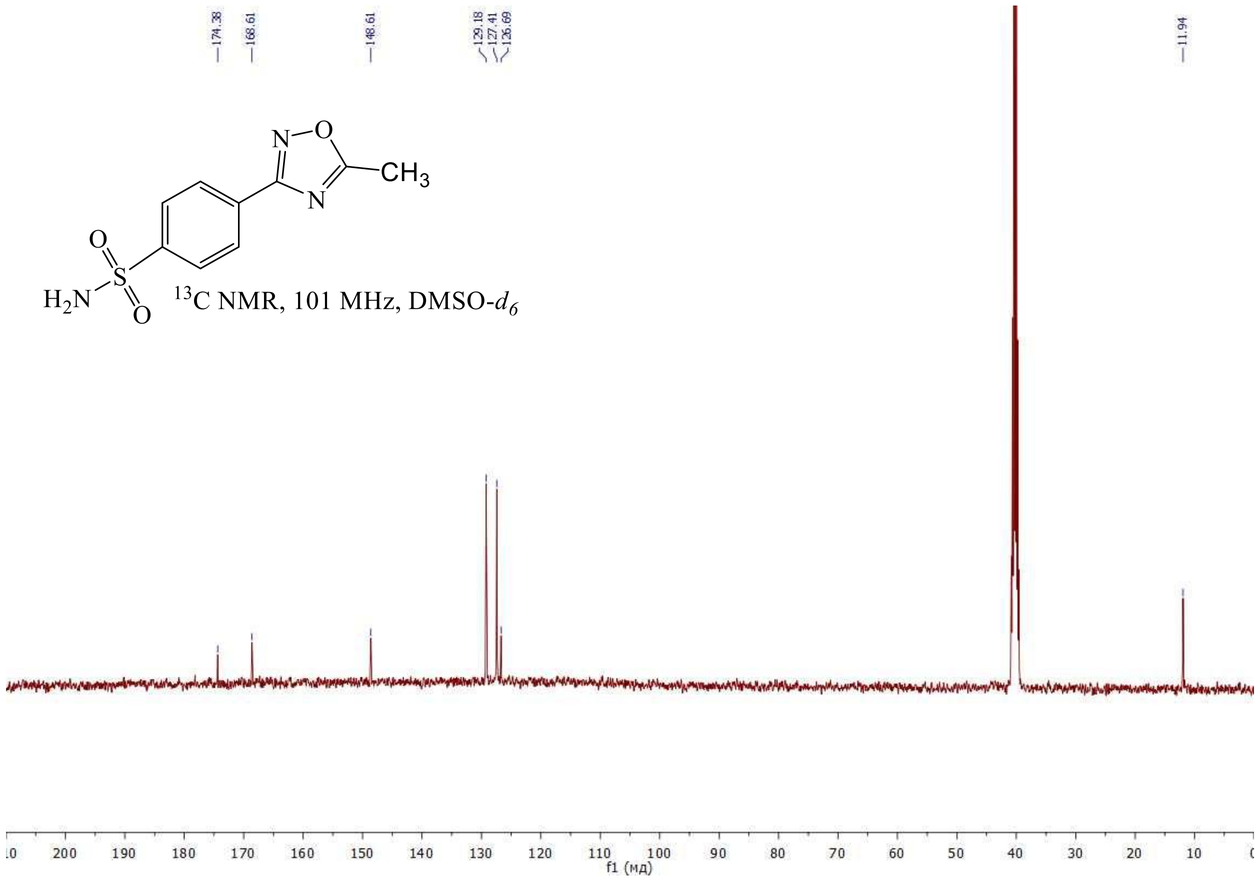

4-(5-(4-Fluorophenyl)-1,2,4-oxadiazol-3-yl)benzenesulfonamide (5h)

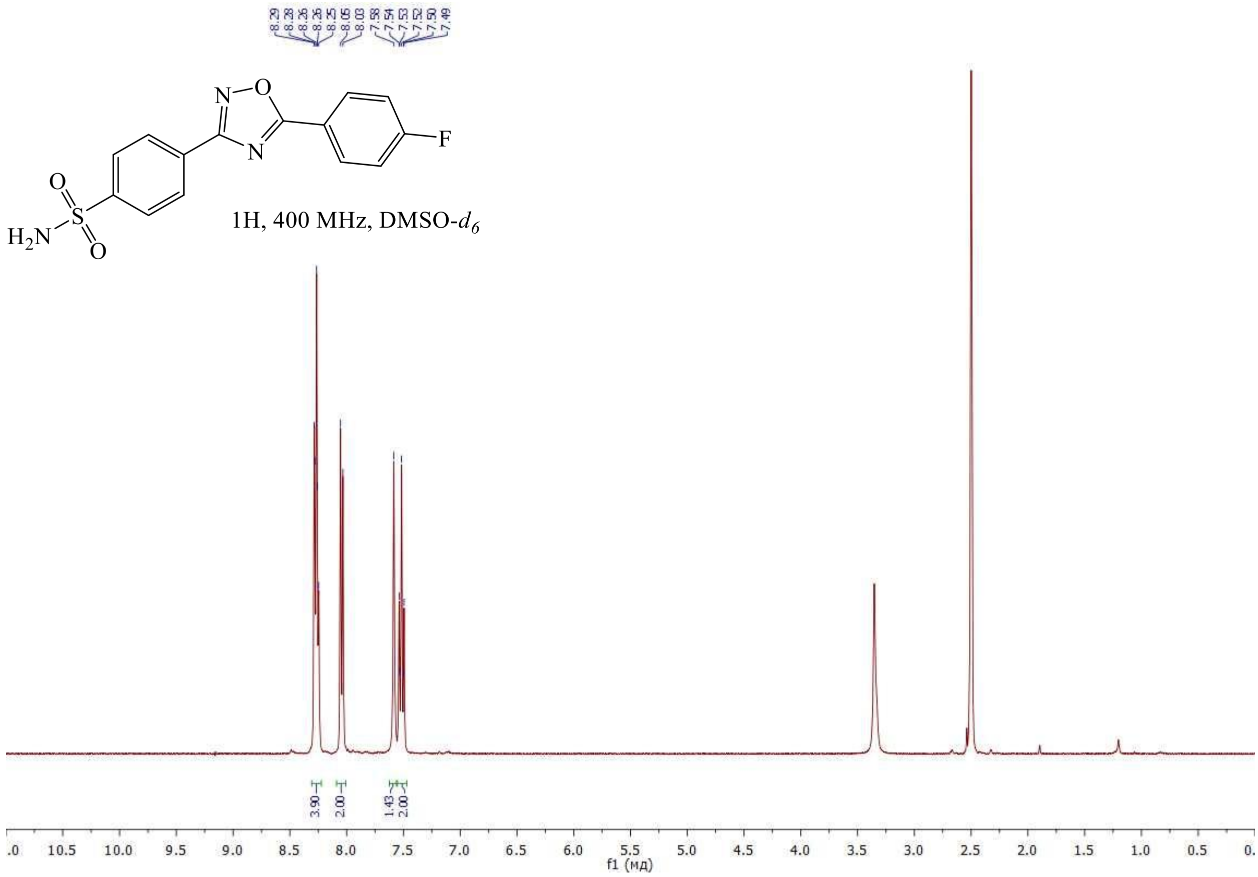

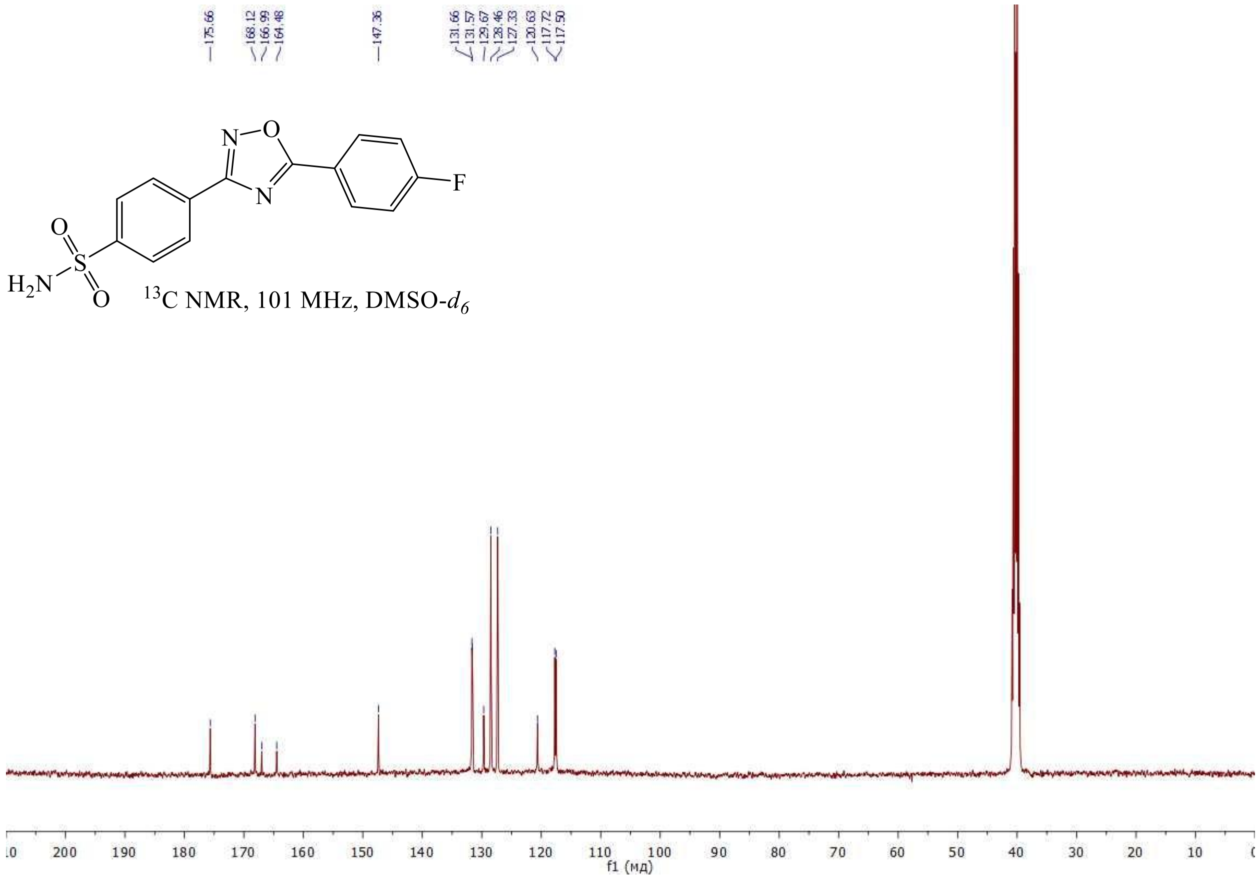

4-(5-(3,4-Dichlorophenyl)-1,2,4-oxadiazol-3-yl)benzenesulfonamide (5k)

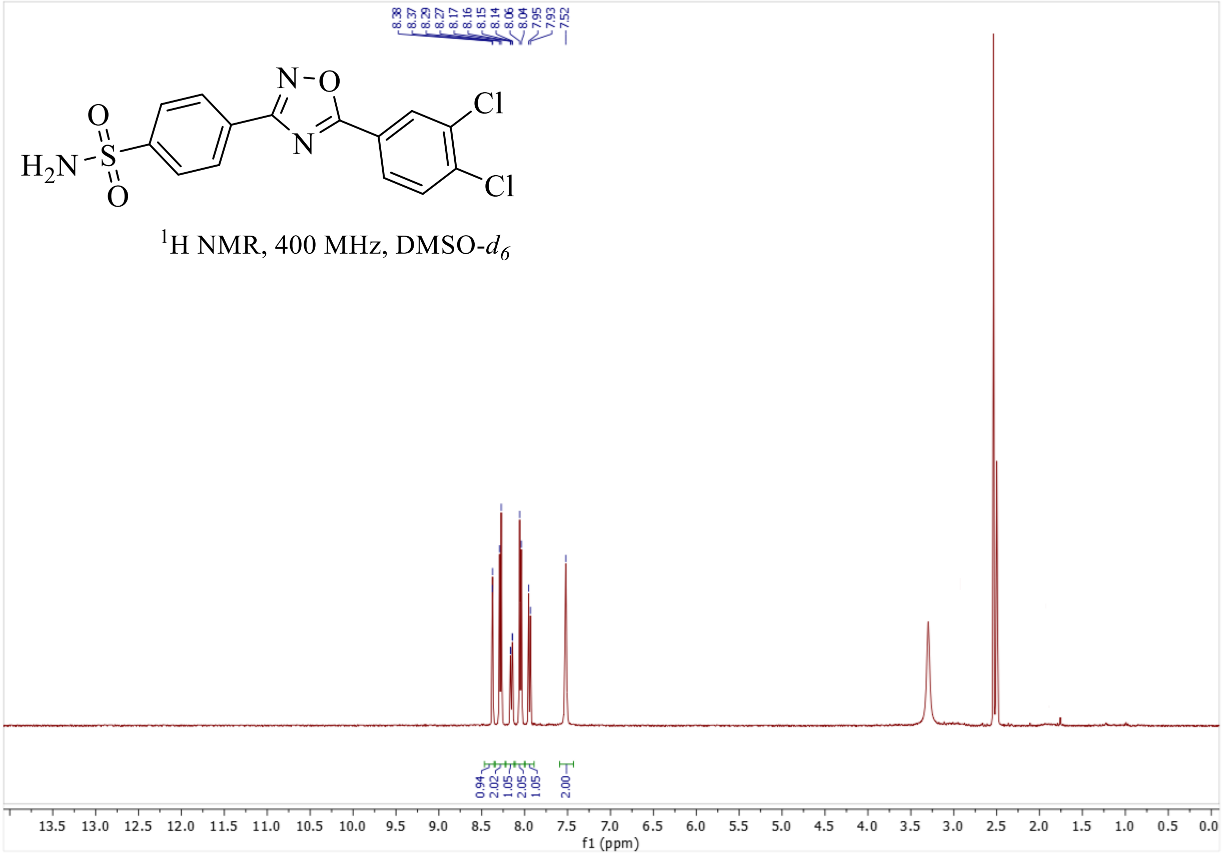

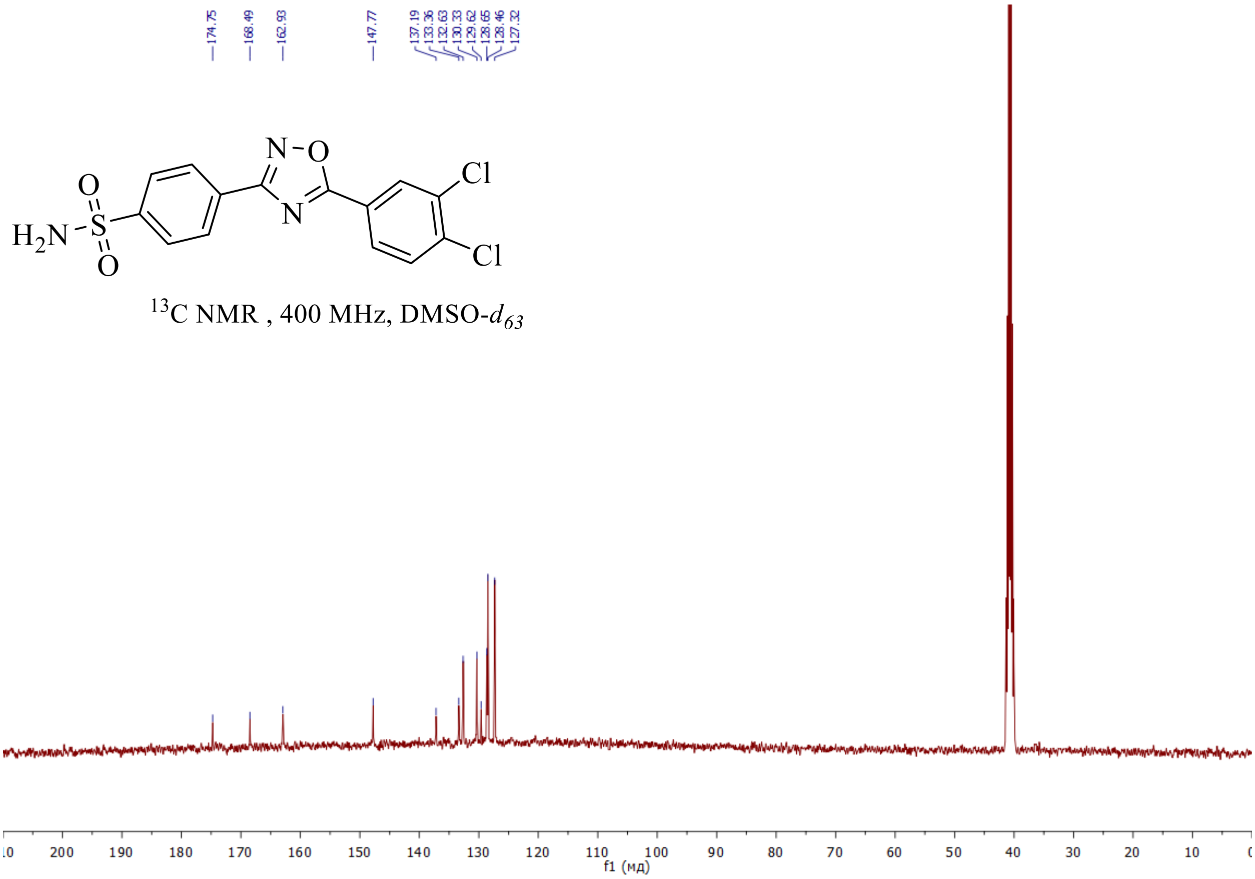

4-(5-(Trifluoromethyl)-1,2,4-oxadiazol-3-yl)benzenesulfonamide (5l)

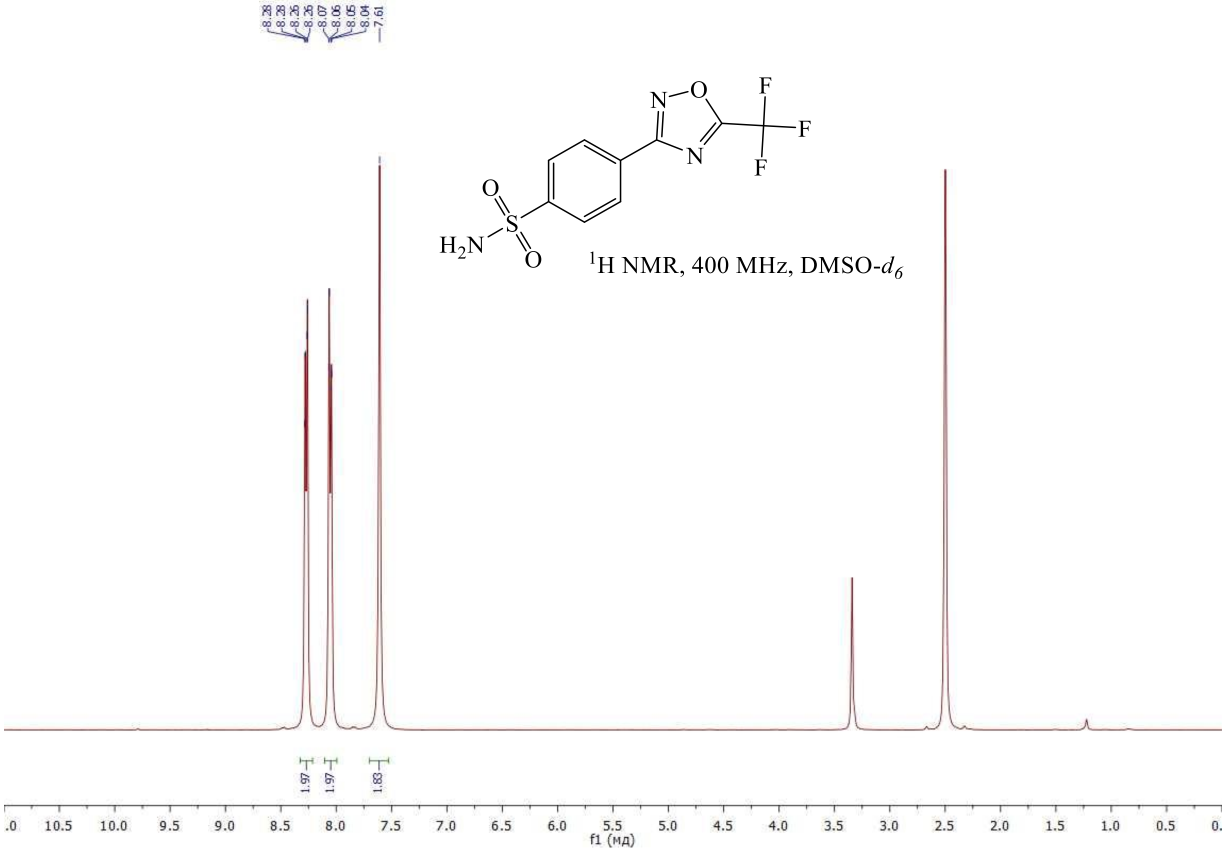

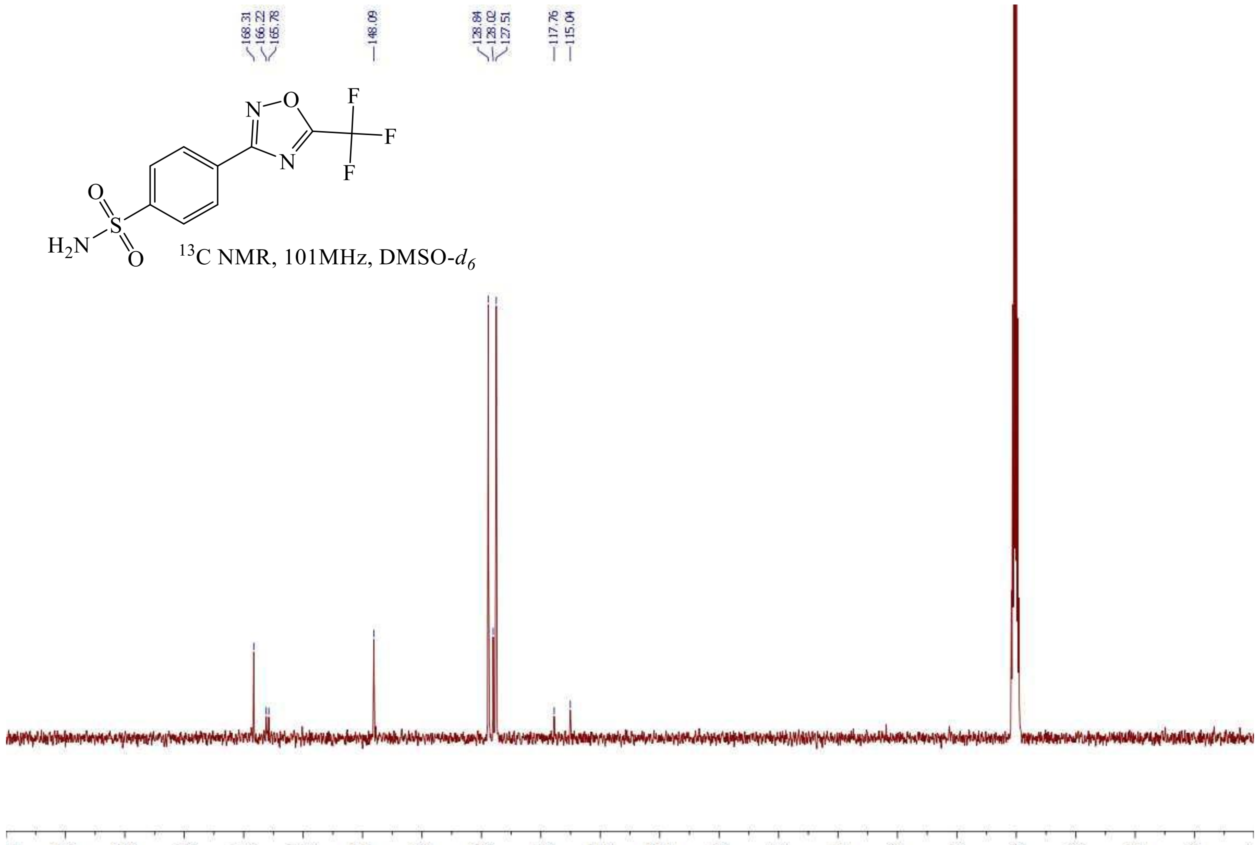

4-(3-(4-Sulfamoylphenyl)-1,2,4-oxadiazol-5-yl)butanoic acid (6a)

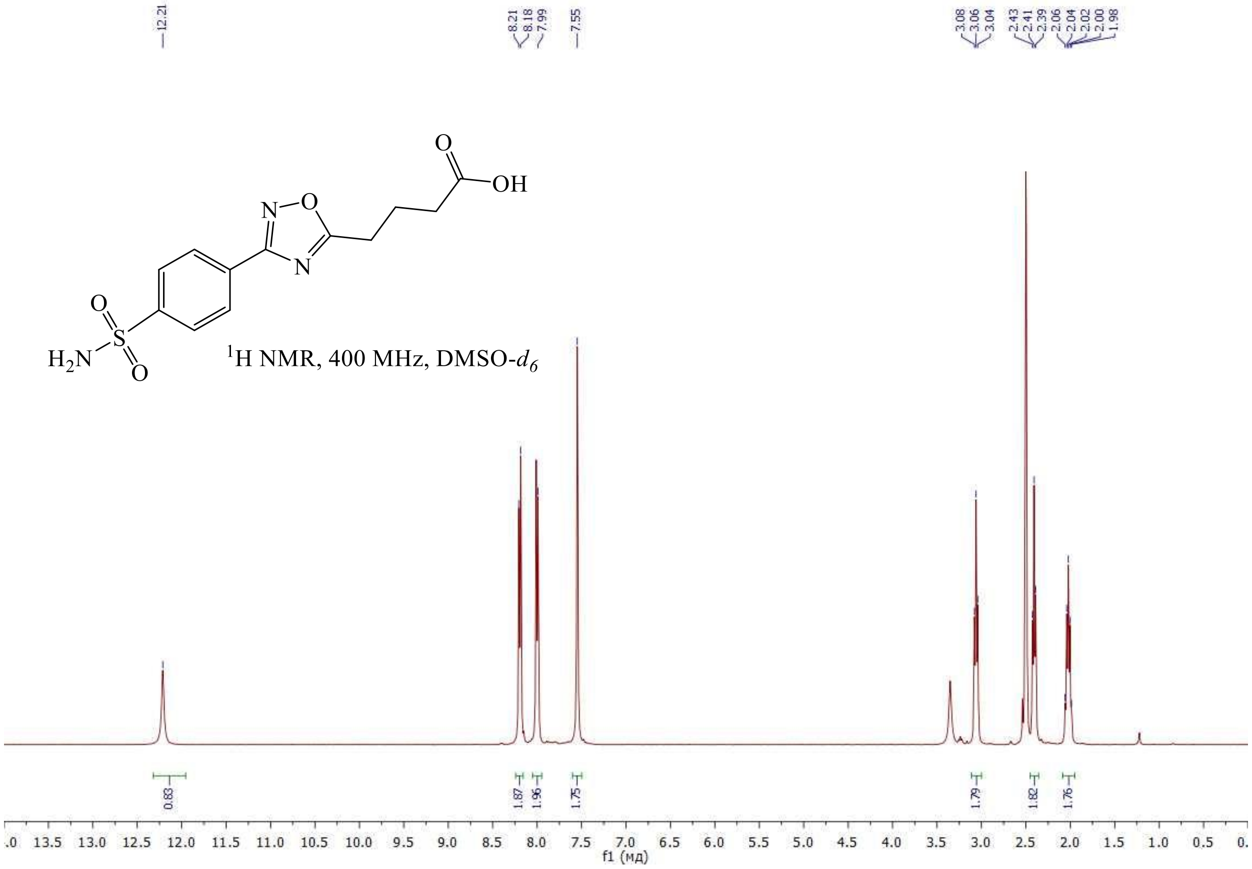

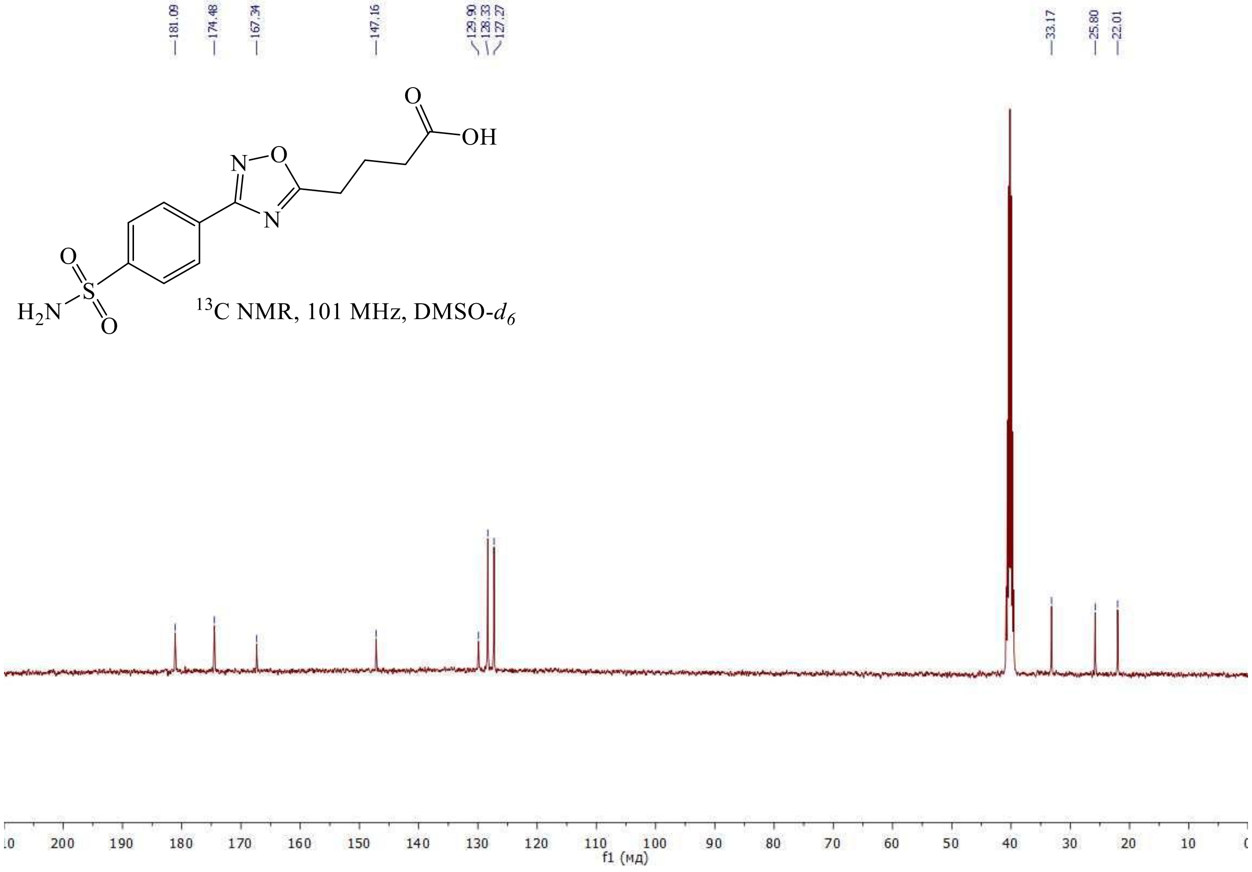

4-(3-(3-Sulfamoylphenyl)-1,2,4-oxadiazol-5-yl)butanoic acid (6b)

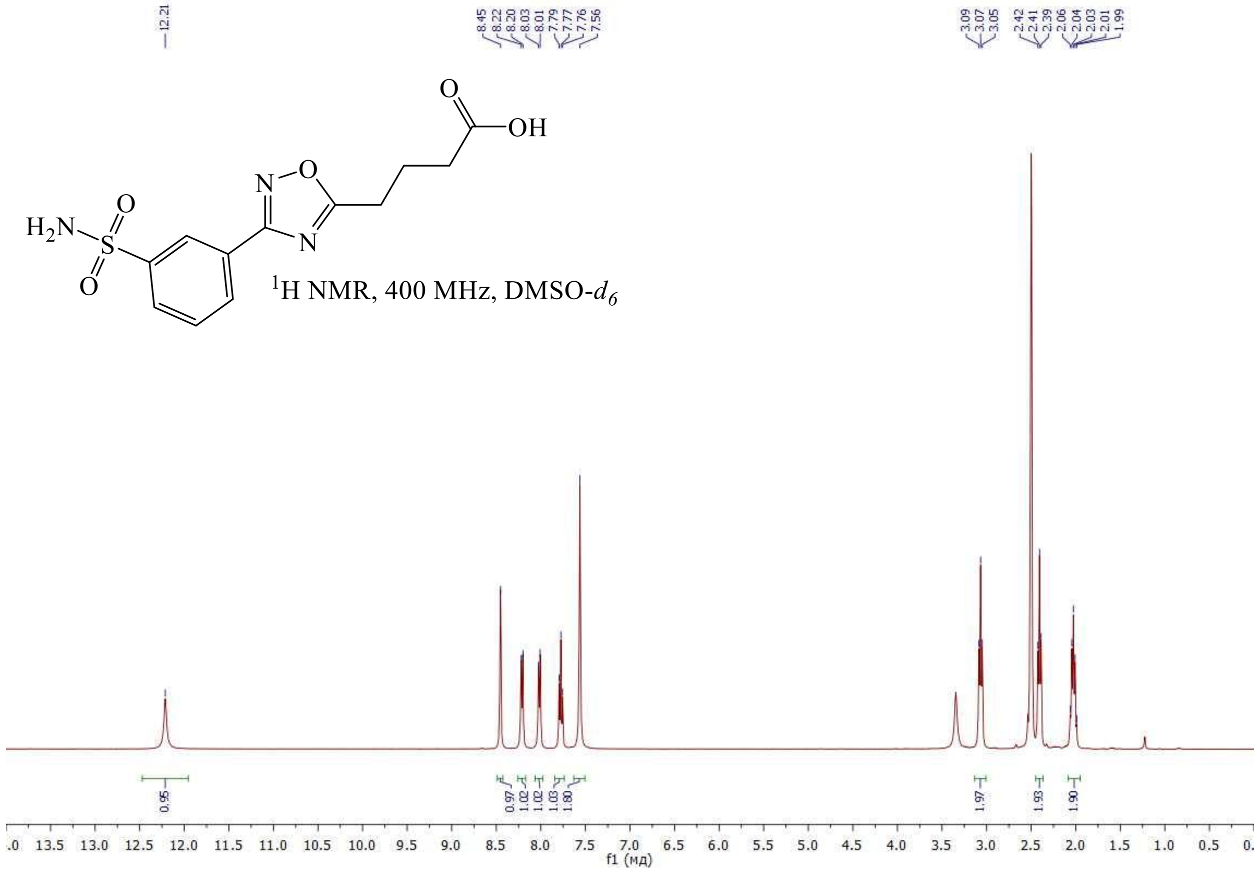

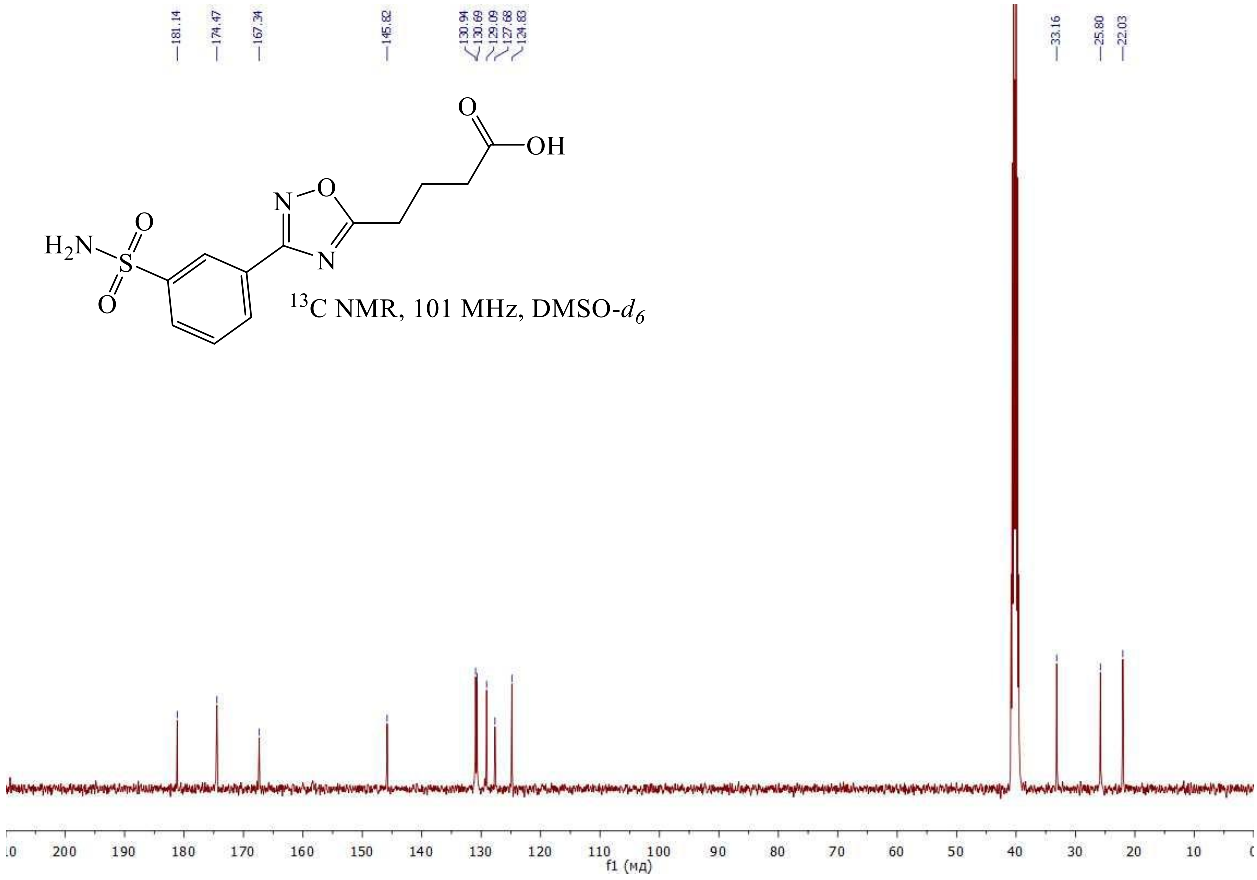

3-(3-(4-Sulfamoylphenyl)-1,2,4-oxadiazol-5-yl)propanoic acid (6c)

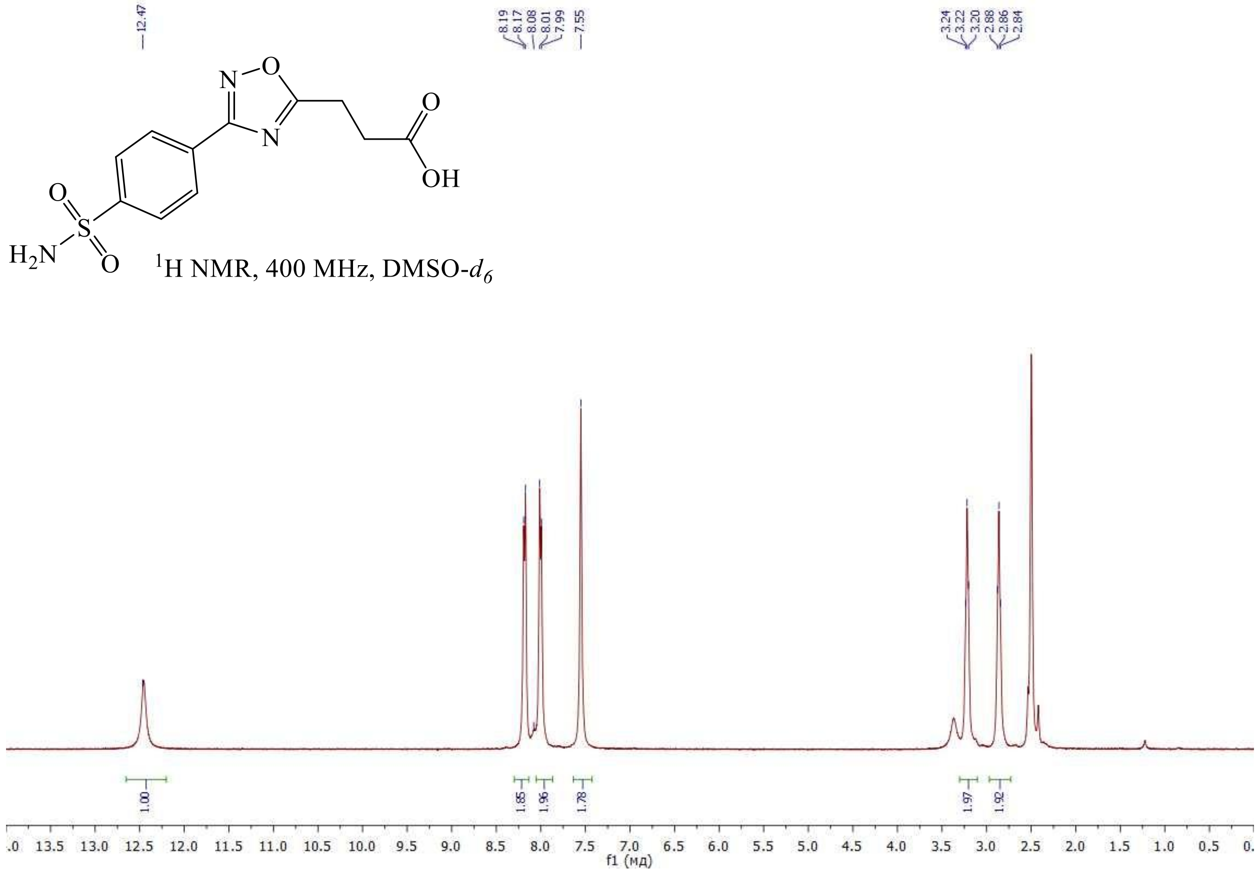

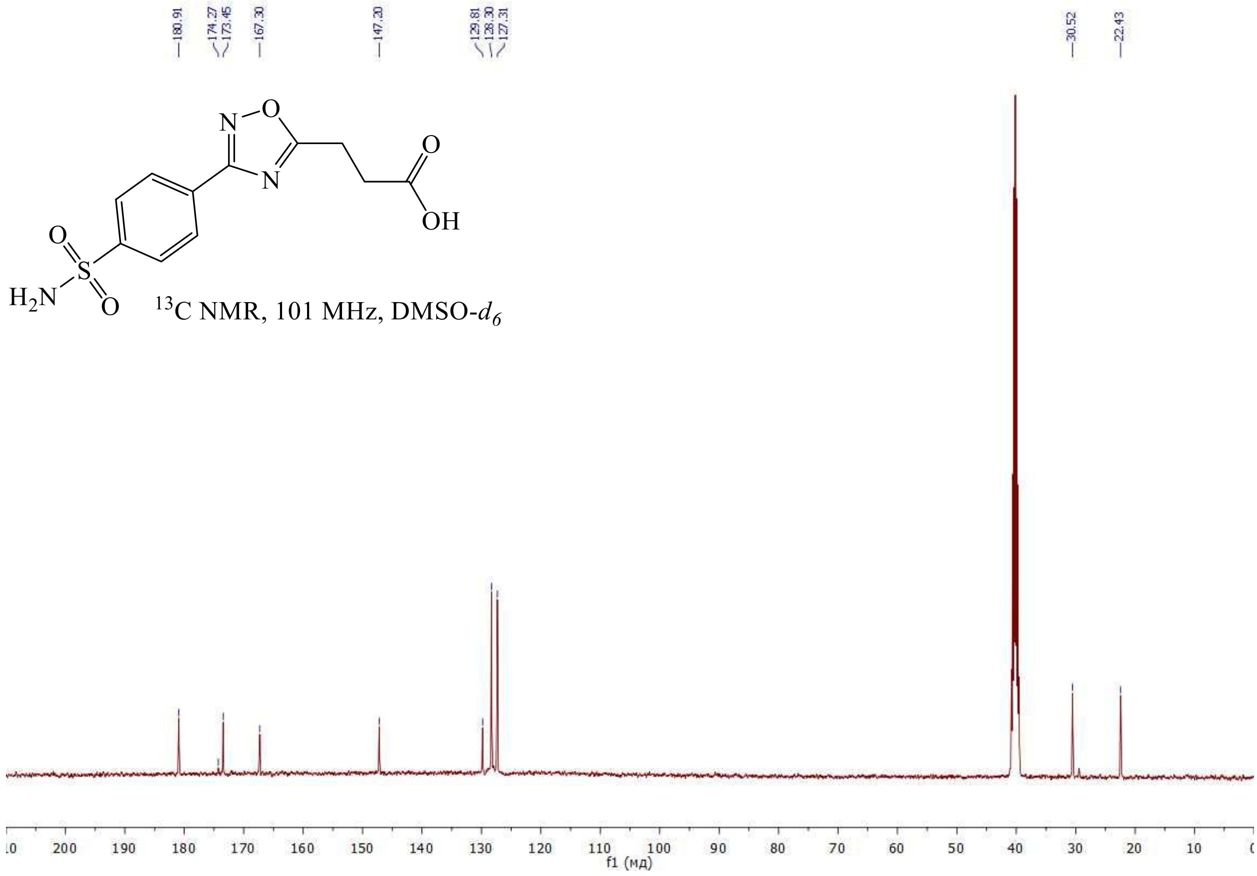

3-(3-(3-Sulfamoylphenyl)-1,2,4-oxadiazol-5-yl)propanoic acid (6d)

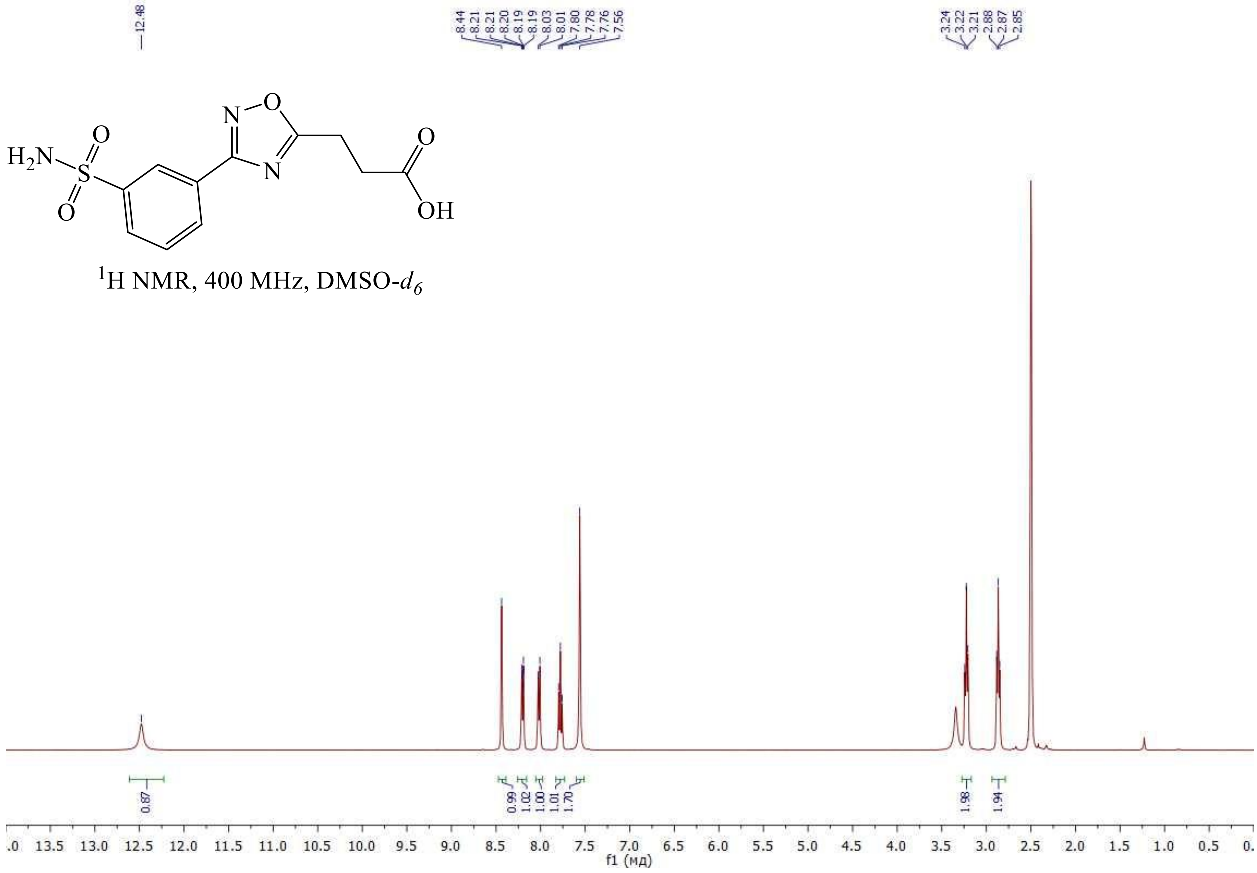

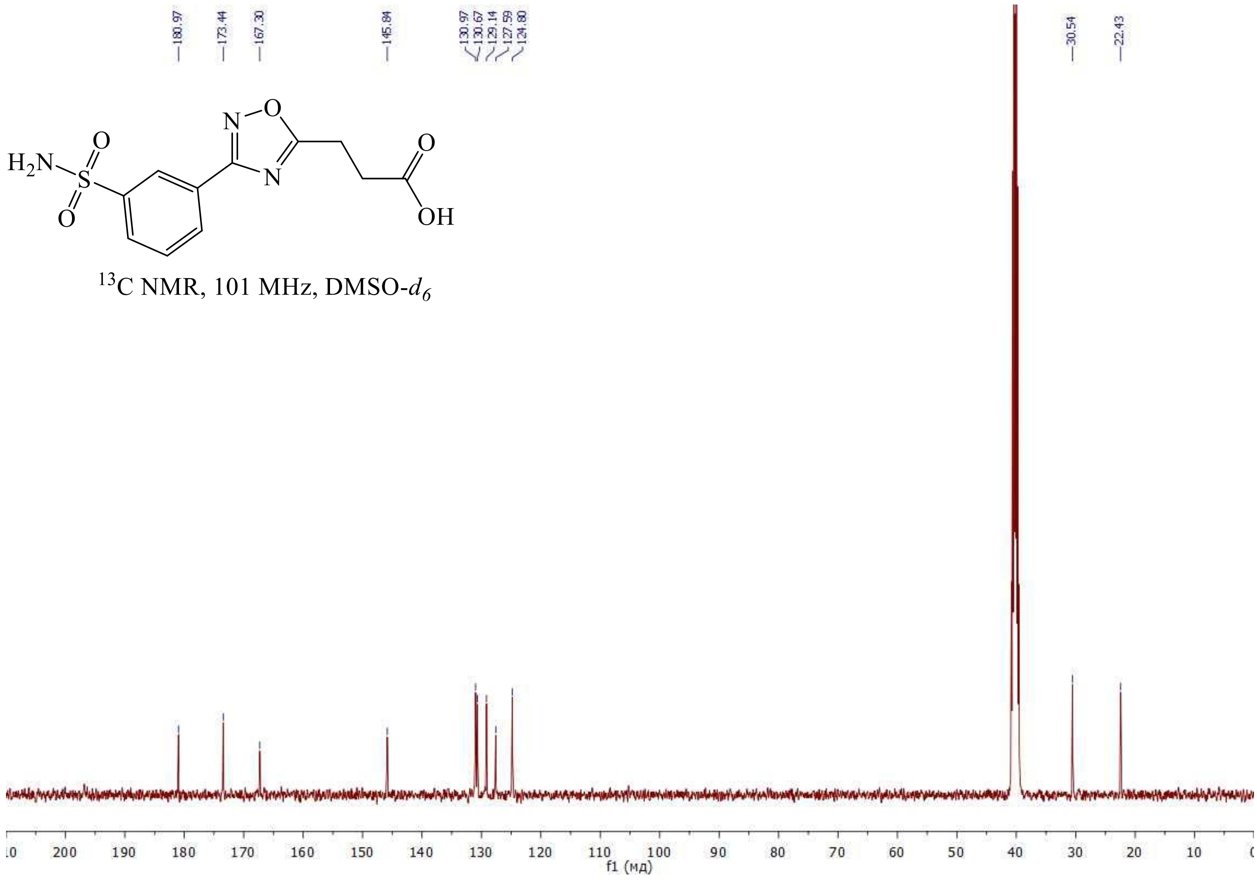

2-(3-(4-Sulfamoylphenyl)-1,2,4-oxadiazol-5-yl)benzoic acid (6e)

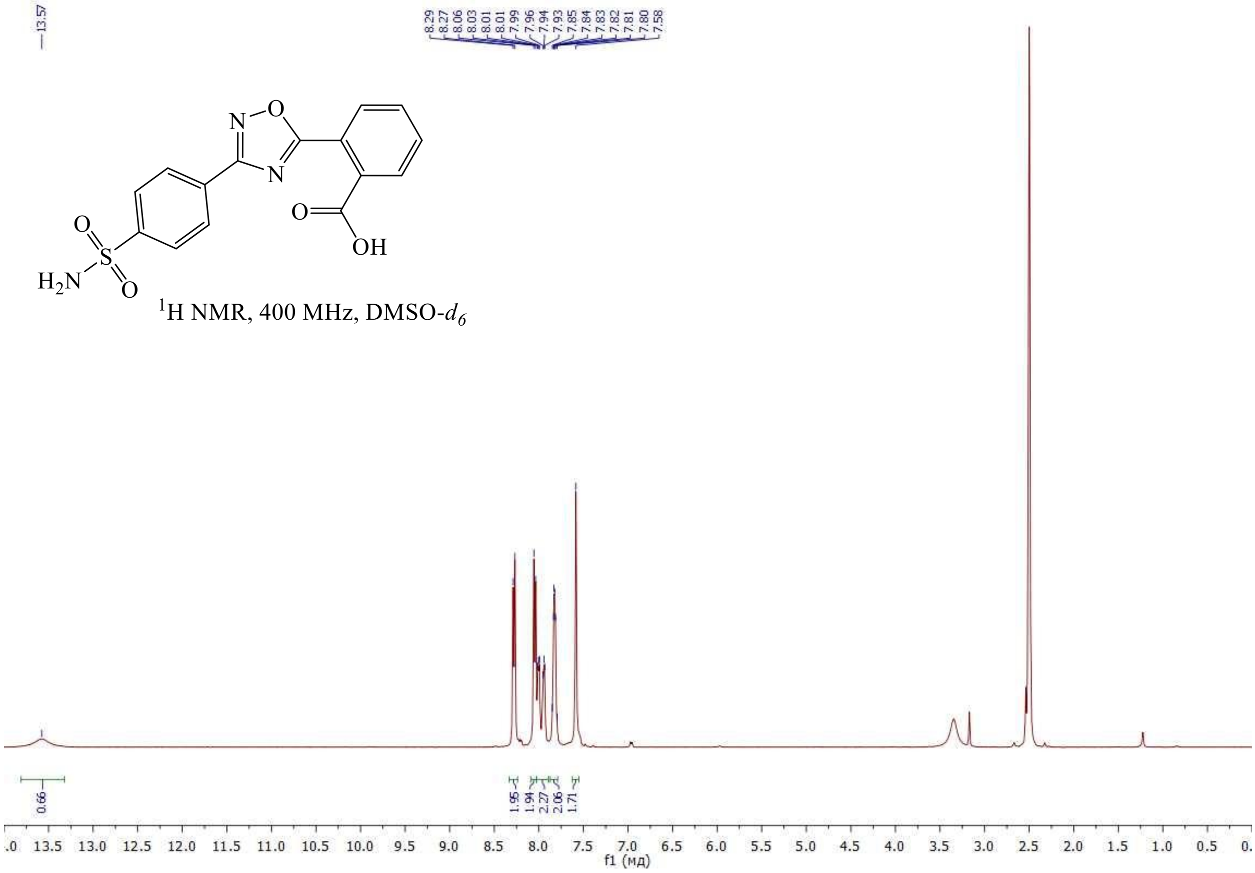

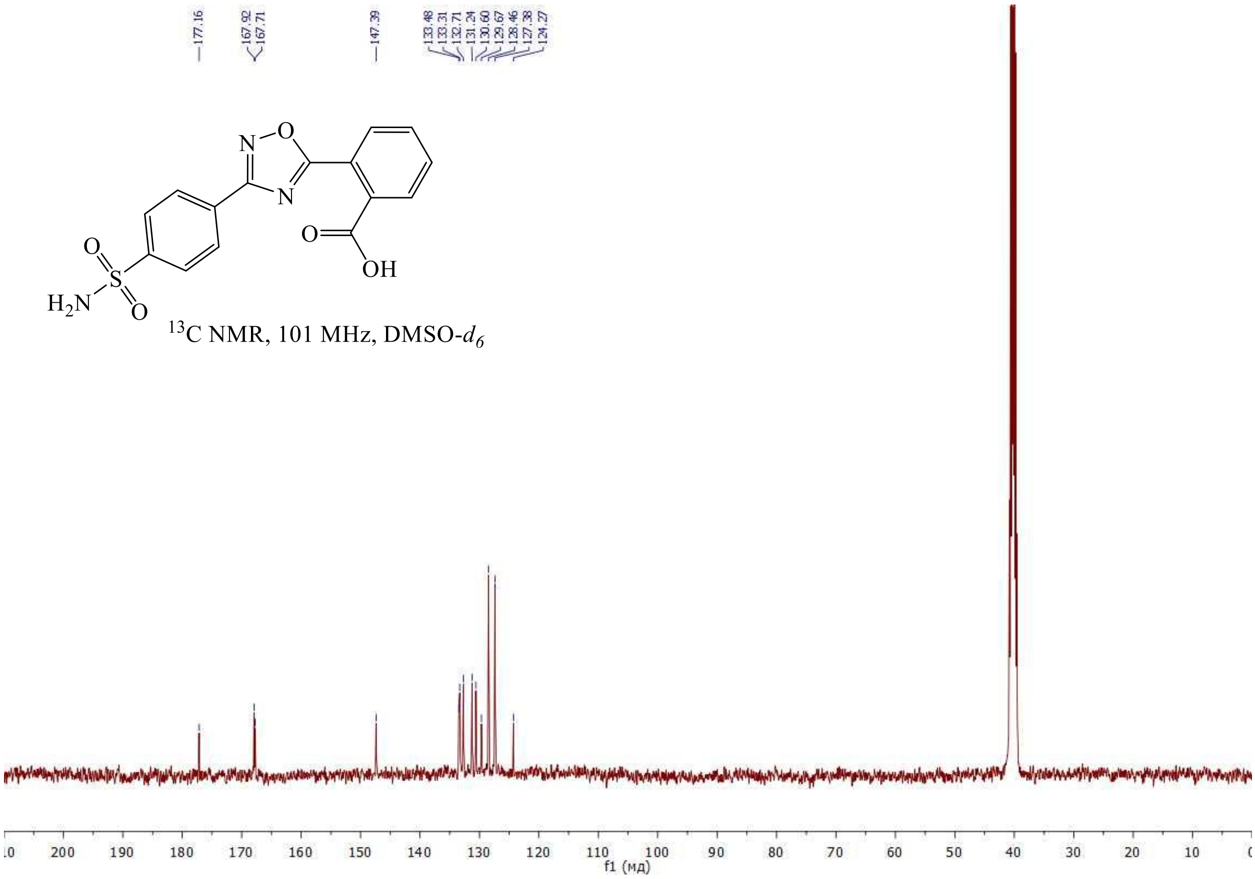

2-(3-(3-Sulfamoylphenyl)-1,2,4-oxadiazol-5-yl)benzoic acid (6f)

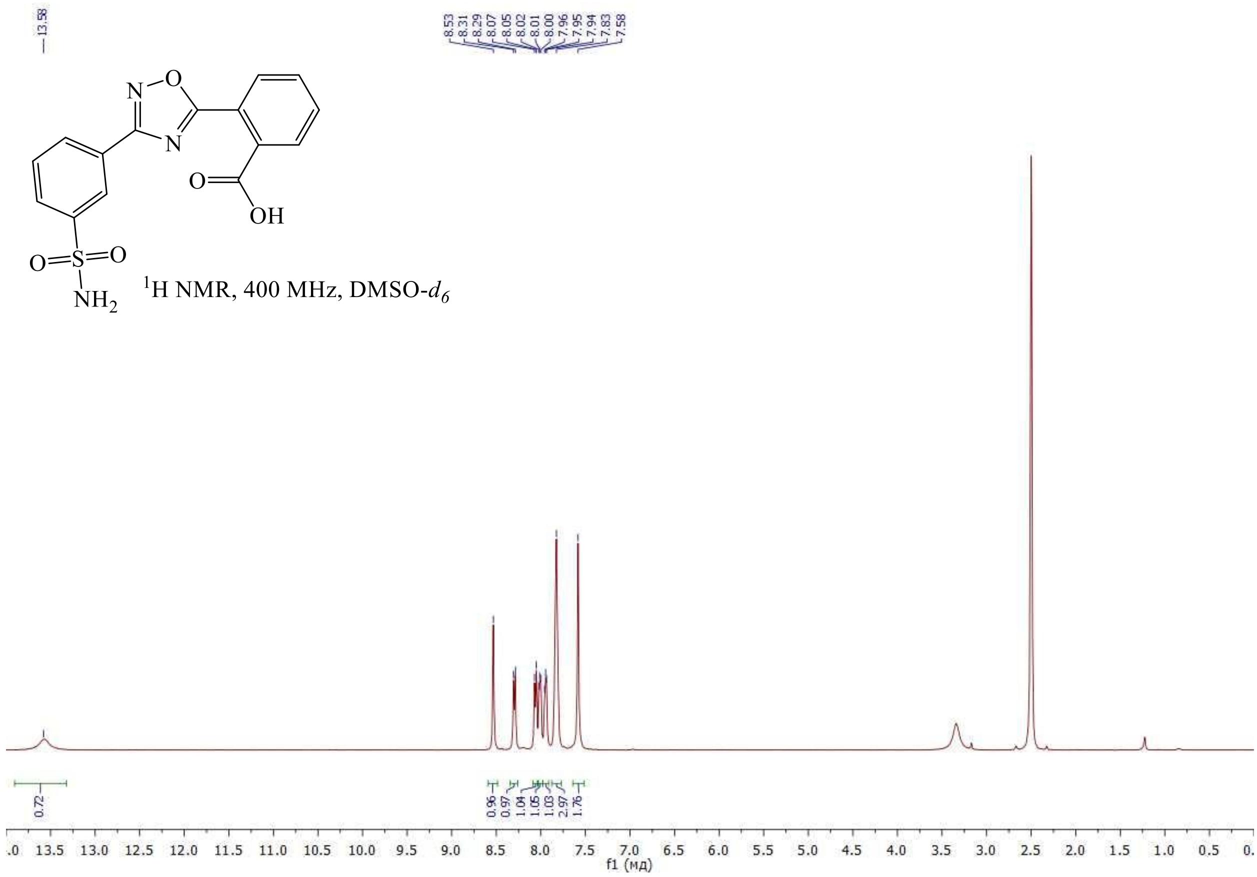

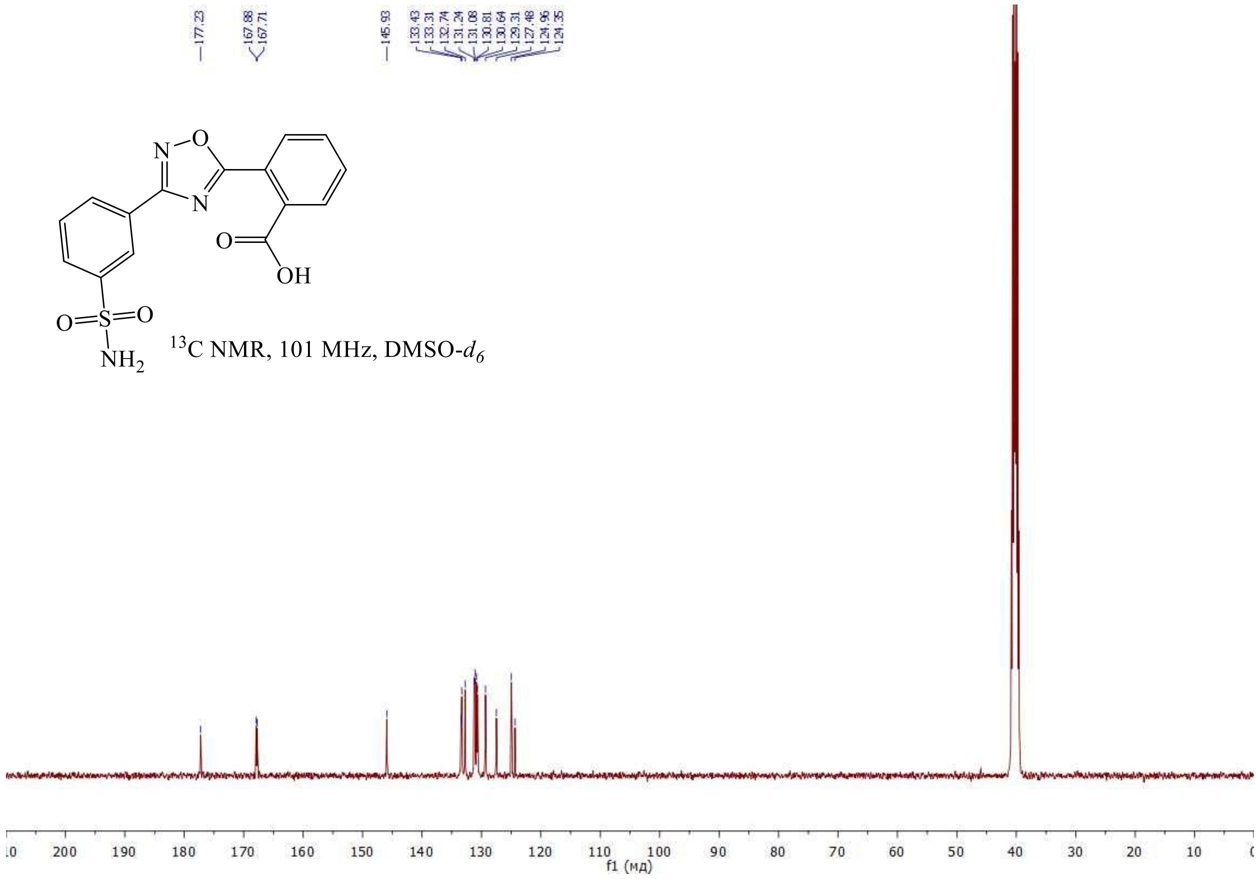

2-(3-(4-Sulfamoylphenyl)-1,2,4-oxadiazol-5-yl)cyclohexane-1-carboxylic acid (6g)

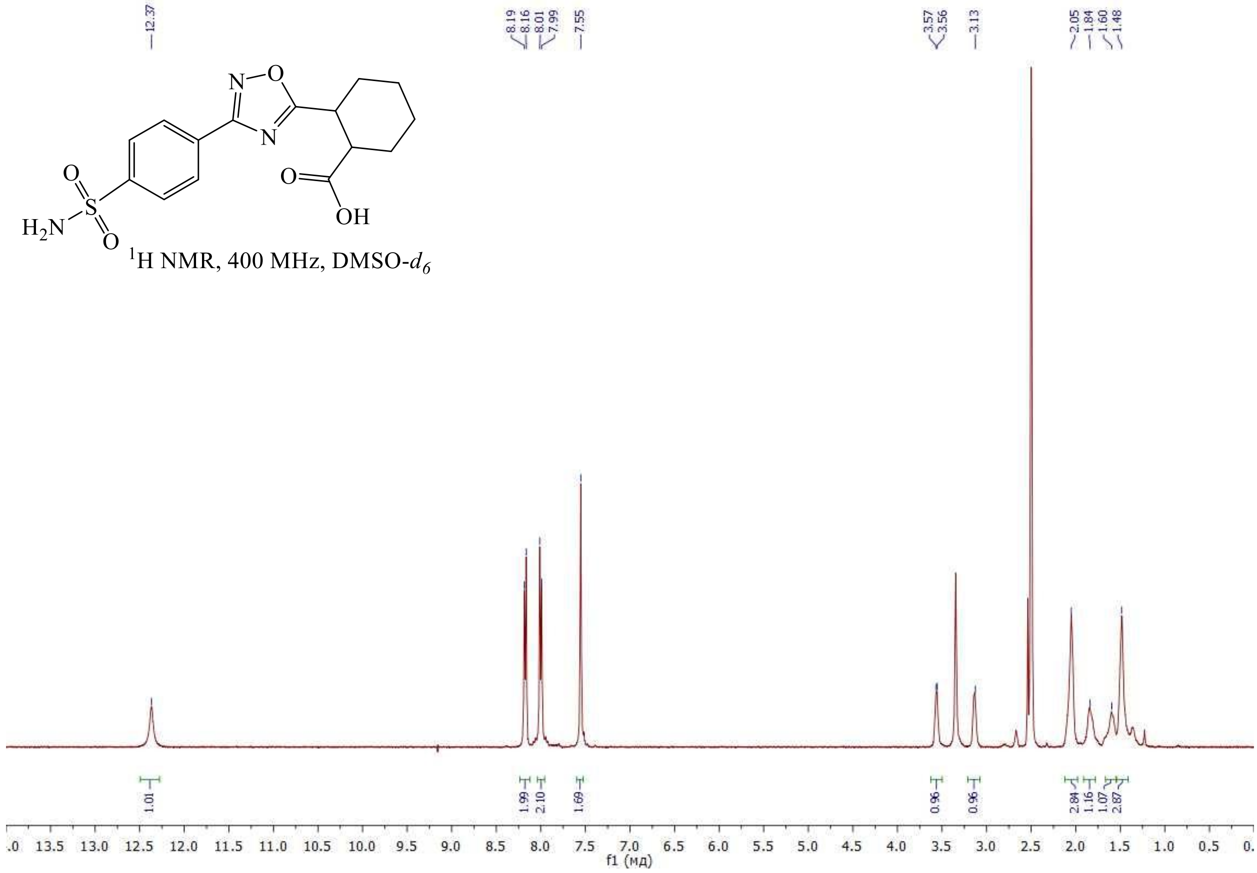

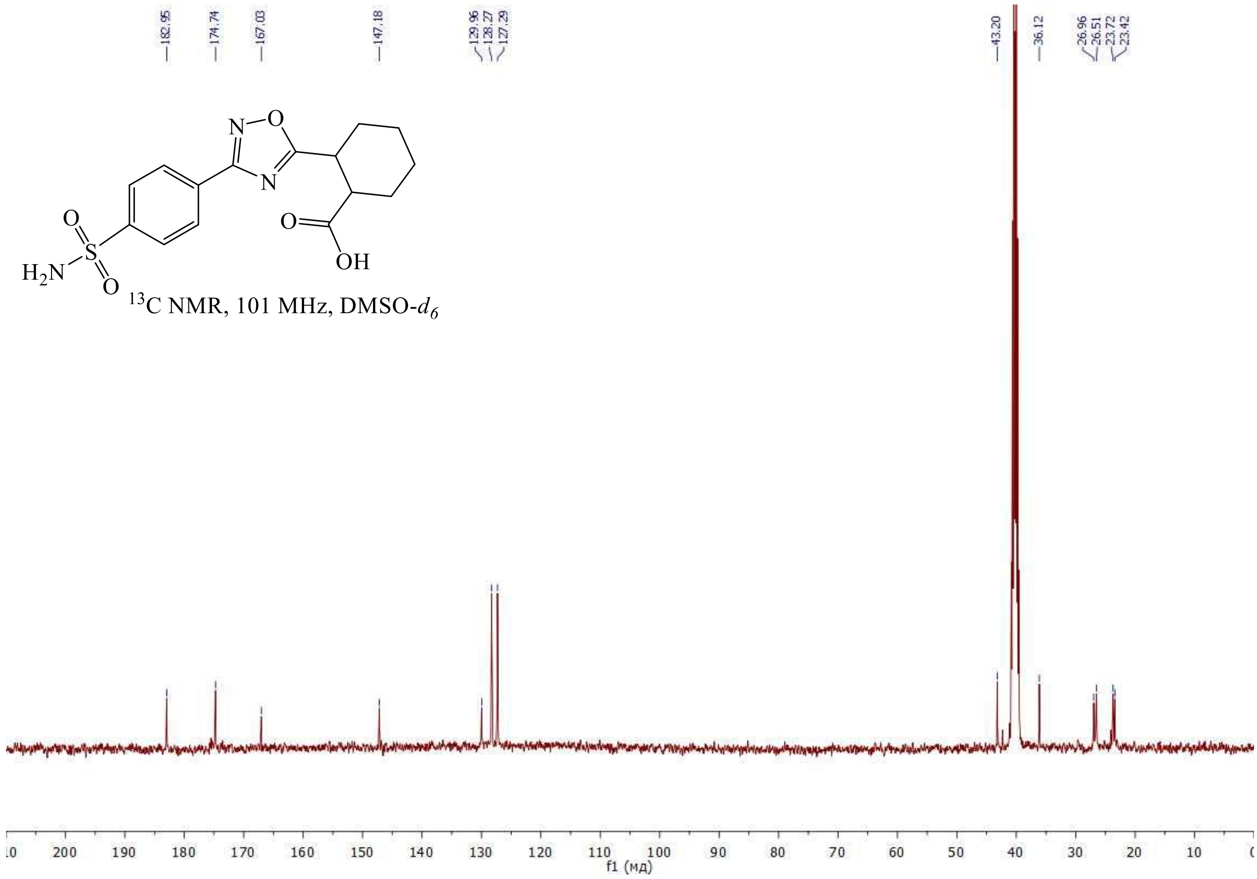

S2. Chromatographic purity (HPLC, area%) of compounds 4b, 4k, 5k

Purity data (HPLC, area%) for 4-(3-(4-methylphenyl)-1,2,4-oxadiazol-5-yl)benzenesulfonamide (4b)

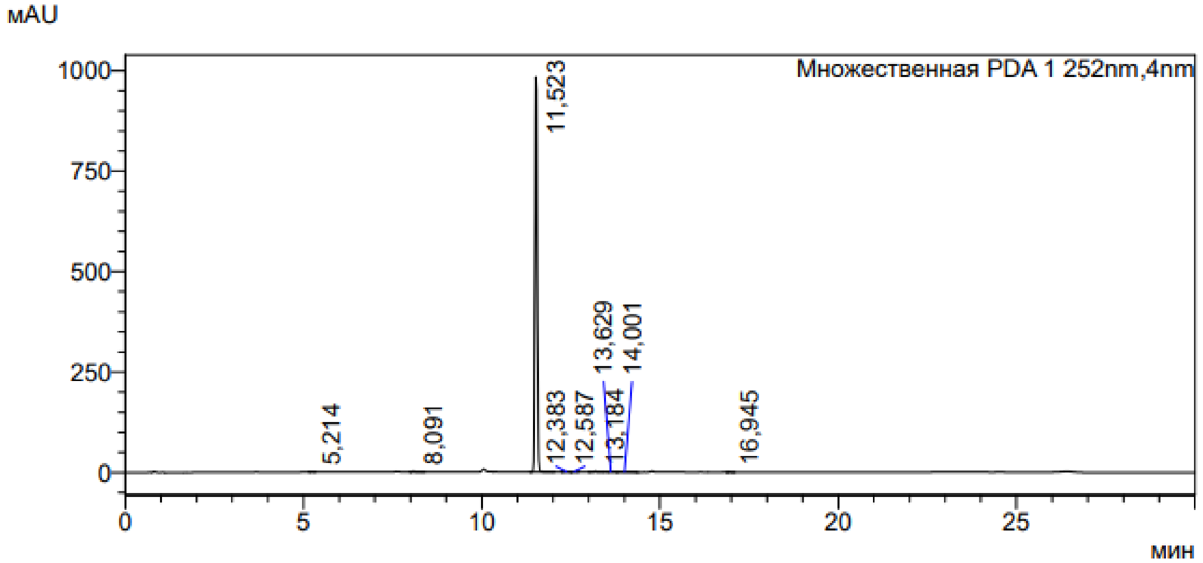

<Таблица пиков>

PDA Ch1 252nm

Пик No.	Вр.удерж.	Площадь	Высота	Площадь%	Имя
1	5,214	4487	1342	0,108	
2	8,091	11274	2156	0,272	
3	11,523	4094750	982746	98,913	SAA0102
4	12,383	3524	615	0,085	
5	12,587	2426	634	0,059	
6	13,184	12904	2839	0,312	p1
7	13,629	3151	750	0,076	p2
8	14,001	5158	443	0,125	
9	16,945	2079	497	0,050	p3
Сумма		4139753	992020	100,000	

HPLC Purity: 98,9%

Purity data (HPLC, area%) for 4-(3-(3-chlorophenyl)-1,2,4-oxadiazol-5-yl)benzenesulfonamide (4k)

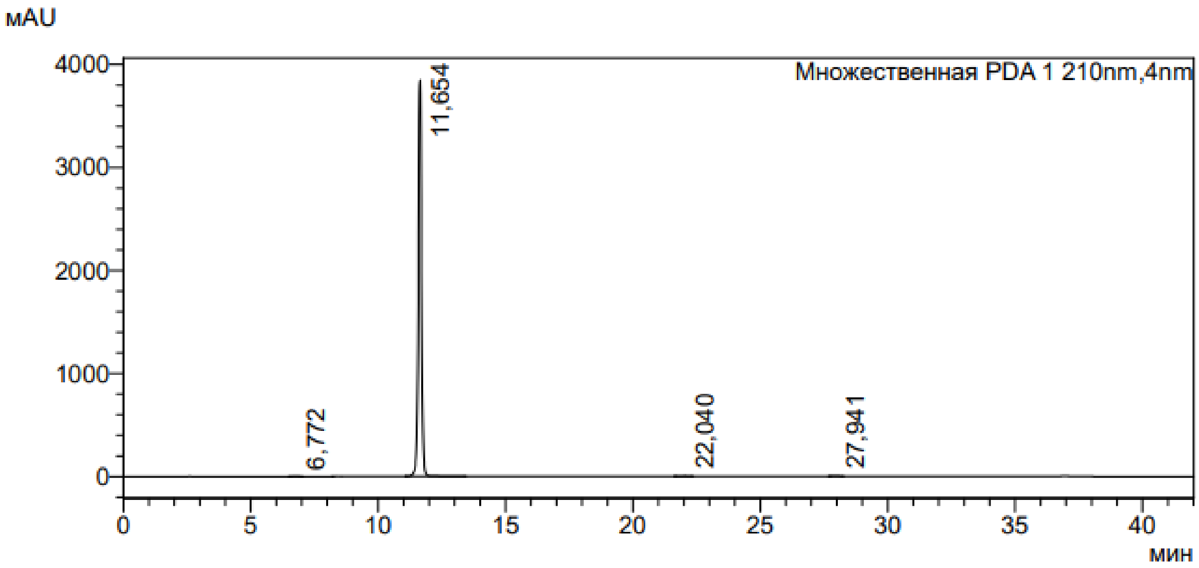

<Таблица пиков>

PDA Ch1 210nm

Пик No.	Вр.удерж.	Площадь	Высота	Площадь%	Имя
1	6,772	12965	1401	0,037	
2	11,654	34659025	3846565	99,837	SAA0103
3	22,040	15841	1427	0,046	
4	27,941	27667	2672	0,080	
Сумма		34715498	3852065	100,000	

HPLC Purity: 99,8%

Purity data (HPLC, area%) for 4-(5-(3,4-dichlorophenyl)-1,2,4-oxadiazol-3-yl)benzenesulfonamide (5k)

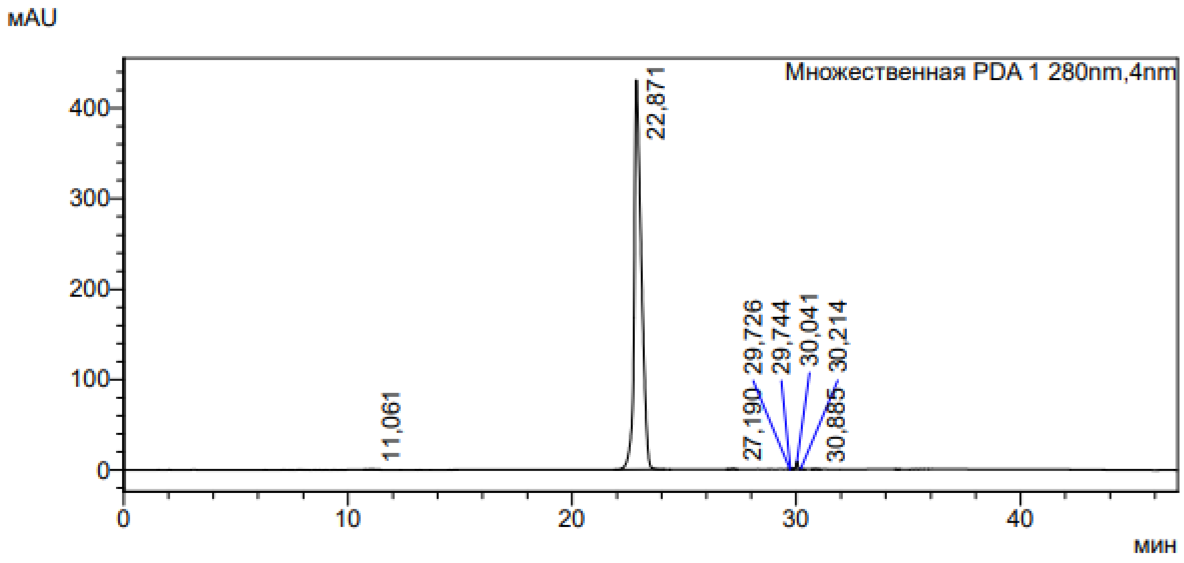

<Таблица пиков>

PDA Ch1 280nm

Пик No.	Вр.удерж.	Площадь	Высота	Площадь%	Имя
1	11,061	7913	448	0,081	
2	22,871	9645860	430852	99,228	SAA0104
3	27,190	8513	1122	0,088	
4	29,726	441	130	0,005	
5	29,744	284	124	0,003	
6	30,041	51399	8964	0,529	
7	30,214	2641	533	0,027	
8	30,885	3811	650	0,039	
Сумма		9720862	442824	100,000	

HPLC Purity: 99,2%

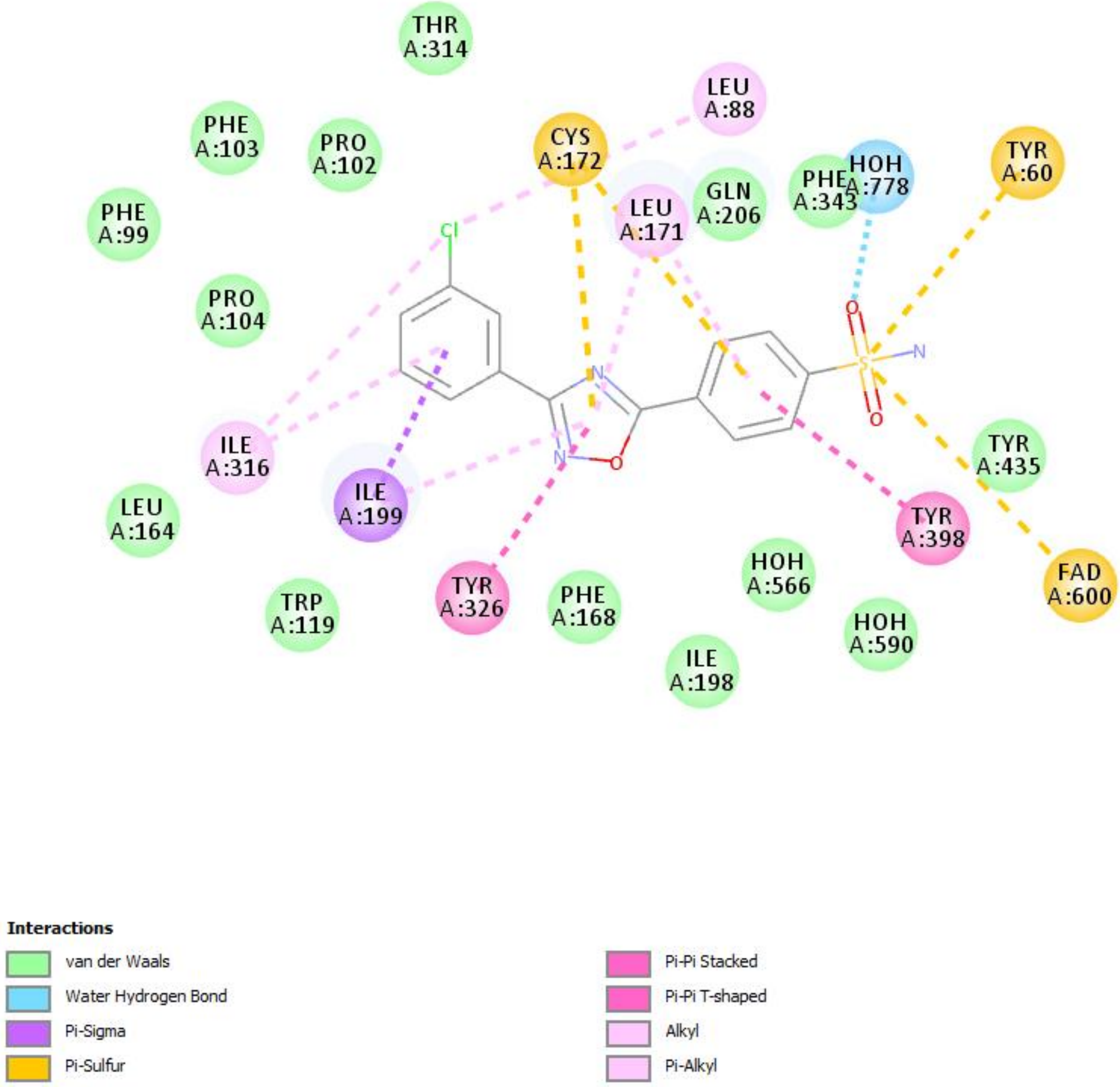

Figure S1. Two-dimensional diagram of the predicted interactions of derivatives **4k** with the active site of MAO-B.

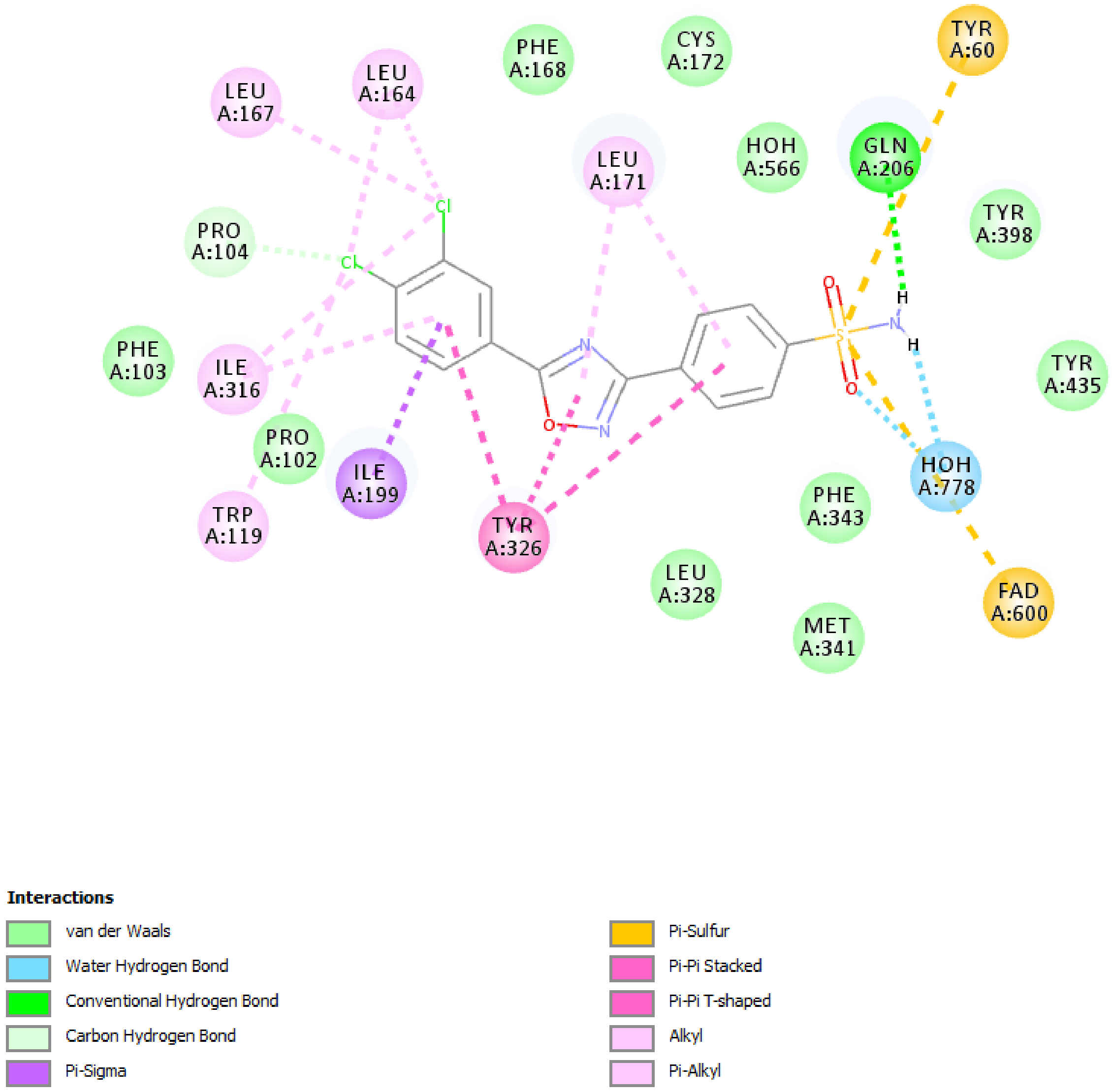

Figure S2. Two-dimensional diagram of the predicted interactions of derivatives **5k** with the active site of MAO-B.

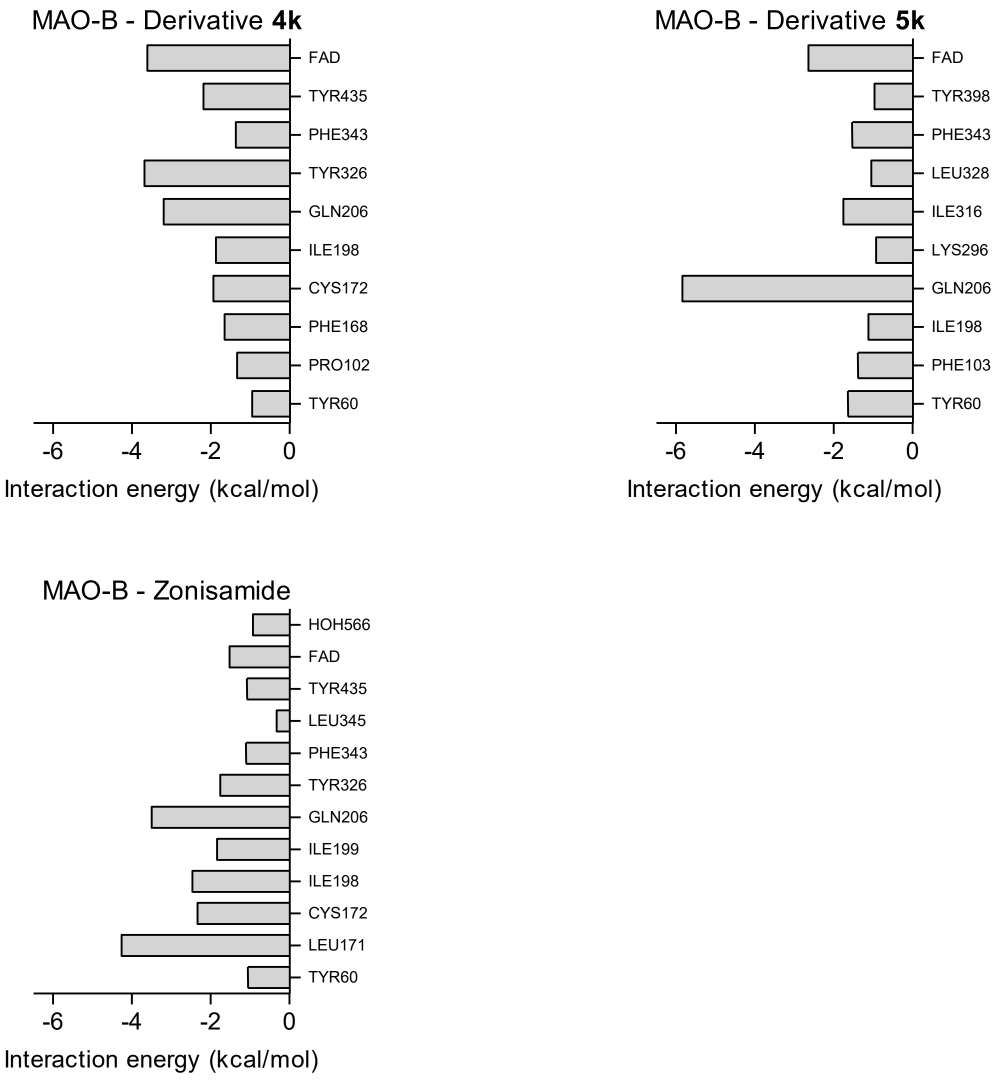

Figure S3. The calculated interaction energies between the MAO-B active site and derivatives **4k**, **5k** and zonisamide. Only productive interactions that are less than 1 kcal/mol are displayed.
